# Progress in the Synthesis of Evidence in Glaucoma: *What We Know and the Gaps that Remain*

**DOI:** 10.1007/s44402-026-00218-w

**Published:** 2026-10-19

**Authors:** Mapa Prabhath Piyasena, Gianni Virgili, Augusto Azuara-Blanco

**Affiliations:** 1 Vision and Eye Research Institute, School of Medicine, Anglia Ruskin University, Cambridge, UK; 2 Centre for Public Health, School of Medicine, Dentistry and Biomedical Sciences, Queen’s University Belfast, Belfast, UK; 3 Department of NEUROFARBA, University of Florence, Florence, Italy

**Keywords:** Blindness, Evidence and Gap Mapping, Evidence synthesis, Glaucoma, Systematic review, Review

## Abstract

**Purpose:**

Glaucoma is one of the leading causes of blindness worldwide, necessitating evidence-informed interventions and strategies to improve the quality of life of affected individuals. In this review, we examined progress in evidence over the past decade and discussed its implications and the ways forward for delivery of glaucoma care globally, embedded within a rapid evidence and gap-mapping exercise of systematic reviews and/or meta-analyses published between 2015 and 2025.

**Methods:**

Using a predefined conceptual framework, ten domains were identified as relevant to evidence synthesis in glaucoma care. Within each domain, we assessed areas of strength and identified remaining evidence gaps. Findings were synthesised narratively and recommendations provided to inform future research priorities and advancement in glaucoma care.

**Recent Findings:**

Across the systematic reviews published over the past decade, the majority focused on risk factors (15.6%), genetic factors (12.4%) and the effectiveness of various treatment modalities (44.4%). In the absence of a coordinated global approach, substantial duplication of effort across review groups was observed. In addition, several critical gaps remain in the evidence base, particularly regarding the cost-effectiveness of the interventions, improving equitable access to care and the availability of context-specific evidence for low- and middle-income countries. Improved rigour in the evidence from primary studies should be a key priority for future research.

**Summary:**

A substantial gap exists in the glaucoma care evidence base that limits its utility for informing clinical guidelines and policy development. Systematic reviewers should have a comprehensive understanding of the existing evidence landscape in glaucoma care prior to conceptualising and designing review projects. Strengthening the methodological quality of both systematic reviews and the underlying primary studies is essential to support the development of robust clinical guidelines and effective global policies in glaucoma care.

Key Points

- Despite global efforts, significant gaps exist in the evidence base of glaucoma care in systematic reviews and meta-analyses, which limit clinical practice guidelines and global policy advocacy.
- Findings suggest more evidence is required regarding the cost-effectiveness of interventions, equity in glaucoma care and the effectiveness of interventions in low- and middle-income countries.
- Strengthening the methodological rigour of systematic reviews and underlying primary research is critical for establishing a robust evidence base for glaucoma care.

## Background

Glaucoma is one of the leading causes of irreversible vision loss worldwide, and its burden continues to increase with population ageing. A recent global meta-analysis estimates that approximately 80.5 million people were living with glaucoma in 2024, with the number projected to rise to nearly 186.6 million by 2060 [1]. In addition to its high prevalence, glaucoma remains a major cause of visual disability. A meta-analysis from the Vision Loss Expert Group estimates that in 2020, glaucoma was responsible for blindness in 3.61 million people and moderate-to-severe vision impairment in a further 4.14 million people globally, accounting for about 8.4% of all blindness worldwide [2].

In the evidence base of vision and eye health, rigorous aggregation of primary studies through systematic reviews and meta-analyses informs day-to-day clinical practice and global eye health policies. Evidence synthesis provides the opportunity to explore the extensive body of data in any field in ophthalmology and optometry. Modern systematic review methodologies emerged from efforts in the mid-twentieth century to synthesise disparate clinical data [3], enabling clinical decisions to be grounded in rigorous, up-to-date summaries of all relevant research, not just selective citations, authorities in the field or traditions [4].

Evidence synthesis through systematic reviews and meta-analyses is distinguished by their transparent methodology, predefined protocols, comprehensive literature searches and critical appraisal of bias. Over time, evidence generated through systematic reviews has become a cornerstone of guideline development, health policy and clinical decision-making worldwide. Thus, it is important to ensure that randomised controlled trials (RCTs) are not underpowered, poorly designed or context-specific; observational studies are not affected by confounding factors and publication bias that may distort the apparent effectiveness of interventions. Evidence synthesis, particularly systematic reviews and meta-analyses, addresses these problems by integrating all available data, increasing statistical power and providing more precise and reliable estimates of effect [5].

Evidence synthesis plays a vital role across healthcare. It informs clinical guidelines, supports shared decision-making with patients, underpins health technology assessment and helps identify gaps in knowledge where further research is needed [6]. This review examines the progress in evidence synthesis over the past decade and its implications for the delivery of glaucoma care.

## Importance of Robust Methodology in Evidence Synthesis

A robust methodology is fundamental to a trustworthy evidence synthesis, helping to ensure transparency, consistency and reduced bias. The Cochrane Eyes and Vision (CEV) group published over 47 systematic reviews and meta-analyses by December 2025 on various aspects of improving glaucoma care [7], making the glaucoma evidence base in the Cochrane Library the second-highest following the domain on retinal diseases. The Cochrane Collaboration’s methodology groups are well-known examples of providing expert guidance regarding high-quality review methods.

Innovative methodology to characterise alternative study designs, other than RCTs, was developed by the Diagnostic Test Accuracy (DTA) Working Group, which led to two large Cochrane DTA reviews specific to glaucoma [8, 9]. In 2015, a large Cochrane review of optical coherence tomography (OCT) for detecting manifest glaucoma included over 100 studies and highlighted the need to move beyond diagnostic case-control designs. These studies overestimated sensitivity and specificity because they compared healthy individuals with patients who had established glaucoma, groups that are more easily distinguished than a consecutive cohort of glaucoma suspects. The review also emphasised the need for longitudinal studies to evaluate better the diagnostic performance of OCT in clinical practice [10]. A further Cochrane review of non-contact tests for detecting angle-closure glaucoma also emphasised the need for high-quality studies to assess the performance of candidate tests in both community-based and secondary care settings [11]. This informed the evidence base for the ACE study (Technologies for the diagnosis of angle-closure glaucoma), a multicentre National Institute for Health and Care Research-United Kingdom sponsored study on the diagnostic performance of OCT and slit-lamp observation of the anterior chamber of the eye to optimise referral of patients with suspected angle-closure glaucoma by community and specialised optometrists to ophthalmologists [12].

## Importance of Outcomes of Evidence Synthesis in Global Eye Health Policy

Robust evidence synthesis is essential for informing global eye health policy. Through the systematic appraisal and integration of available research, it provides a reliable foundation for policy development, resource allocation and the implementation of evidence-based interventions to improve glaucoma care and visual health outcomes worldwide. One example is the contribution of the CEV group to the development of the World Health Organization (WHO) *Package of Eye Care Interventions* (PECI), an evidence-based resource that outlines recommended eye care interventions across the continuum of care, together with the material resources required for their implementation [13].

The PECI is intended to support policymakers and technical decision-makers, particularly in low- and middle-income countries (LMICs), in integrating eye care into national health policies and service packages. It also provides guidance for service providers in planning and implementing eye care programmes and serves as a reference for donor and development agencies when designing and supporting eye care initiatives. CEV contributed methodological expertise to the WHO by identifying and appraising high-quality clinical guidelines and systematic reviews to inform the development of the PECI. The PECI also highlights the substantial resource constraints affecting glaucoma care in many LMICs. For example, a national survey of glaucoma practice patterns in Nigeria found that only around 30% of ophthalmologists had access to laser equipment, while 15–20% of clinics lacked basic diagnostic equipment required for glaucoma diagnosis and management [14].

## Research Priorities in Glaucoma Care

Currently, there is no coordinated global strategy to strengthen the evidence base for glaucoma care, increasing the risk of duplication of research efforts and inefficient use of limited research resources. A priority-setting exercise conducted by the CEV Priority Setting Group identified evidence synthesis for paediatric eye care and rehabilitation as major gaps, priorities that are also relevant to glaucoma research [15].

Participants in the Delphi consensus process emphasised the importance of disseminating evidence synthesis findings in formats that are accessible to a broad range of stakeholders. They also highlighted the need to report outcomes disaggregated by sex, gender and ethnicity to support equitable eye care delivery. The priority-setting exercise further identified several evidence gaps specific to glaucoma, including monitoring strategies to prevent disease progression, interventions to improve referral pathways, the use of telemedicine/telemetry for remote patient monitoring, applications of artificial intelligence (AI) in glaucoma care, the diagnostic and monitoring role of OCT and prognostic factor reviews [16]. Questions related to monitoring glaucoma and improving the referral pathway, including telemetry, AI and OCT, were ranked highest.

## Evidence and Gap Mapping Exercise for Glaucoma

Identifying the gaps in the current evidence base is essential to guide future primary research, inform clinical guidelines, policy formulation and prioritise scarce resource allocation. An Evidence and Gap Mapping (EGM) exercise through an overview of published systematic reviews provides a structured approach to synthesise and critically evaluate the evidence base and map the gaps that exist. We conducted a rapid EGM exercise embedded in this review to understand which areas of glaucoma care are well supported by high-quality systematic reviews (±meta-analysis) and where evidence gaps remain.

We followed EGM methodologies using the steps described by Khali et al. [17] i.e., (1) Define review scope, (2) Develop a protocol, (3) Searching the literature (Supplementary File 1—Electronic database search strategy), (4) Screen studies, (5) Data extraction, (6) Data coding and categorisation, (7) Risk of bias assessment [optional], (8) Utilise visual presentation of the data to facilitate analysis and usability of the review, (9) Interpret and discuss findings and (10) Report the findings [17]. This EGM methodology enabled a comprehensive, transparent characterisation of the glaucoma evidence base over the last decade. In the Population-Concept-Context framework we used: *Population*—patients with any type of glaucoma or at risk of developing glaucoma; *Concept*—How to improve glaucoma care, leading to controlling vision impairment and blindness in the population (screening, diagnosis, treatment and rehabilitation) and the *Context*—Globally in any country/income setting.

**Fig. 1:**
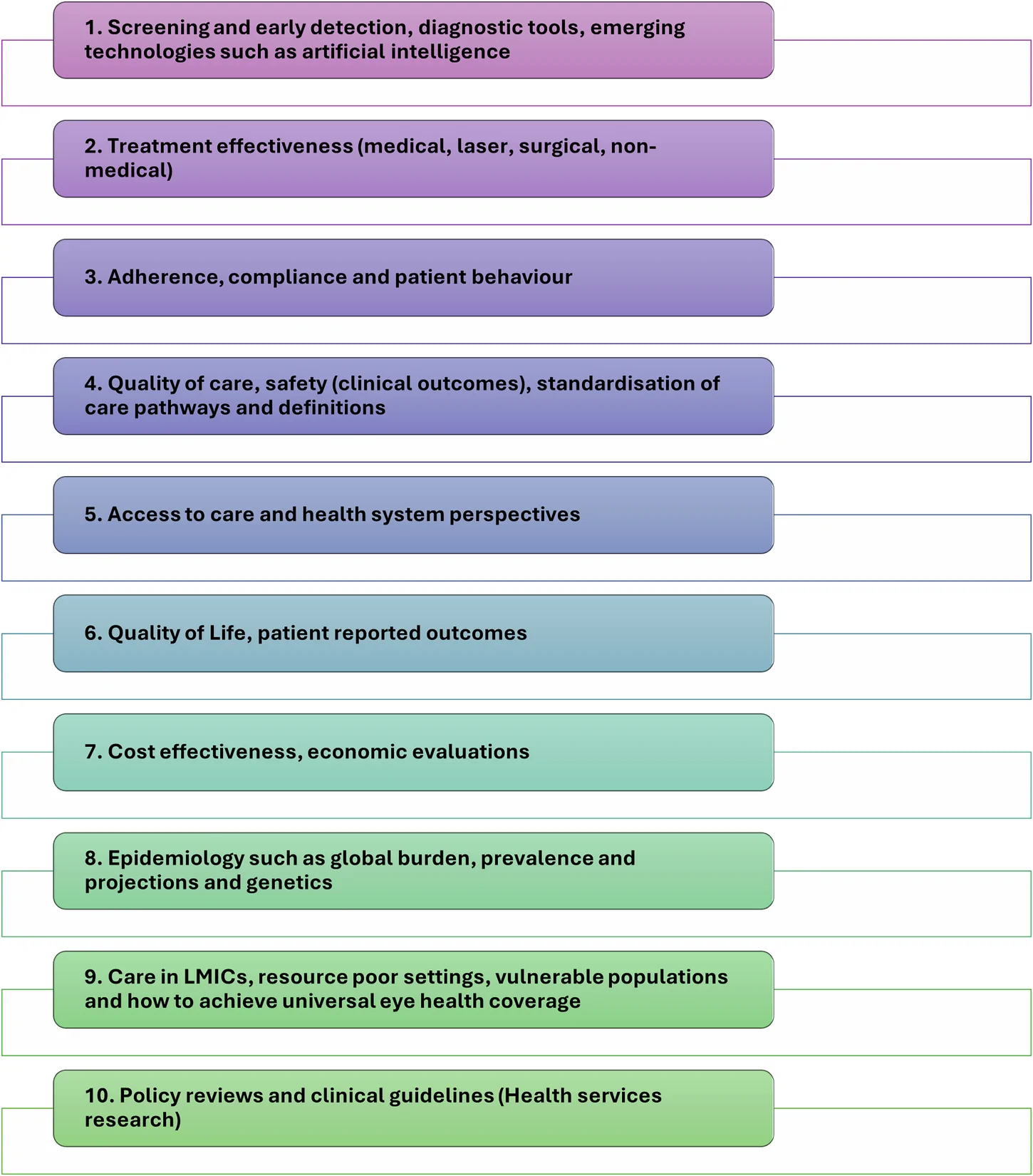
Conceptual framework for the Evidence and Gap Mapping exercise. LMICs low- and middle-income countries.

We included only systematic reviews and meta-analyses in this EGM exercise. We searched through MEDLINE All (Ovid) and Cochrane CENTRAL CDSR from 1st January 2015 up to 31st December 2025. In the absence of an established framework, we designed a novel classification that spanned epidemiology, clinical management, quality of life and health policy. In our conceptual framework of EGM, we decided that at least the following 10 domains should be considered in evidence synthesis in glaucoma. These are given in Fig. 1, starting from screening to rehabilitation and including health system and epidemiology perspectives. Because each domain was assigned equal weights in our EGM, a hierarchical ranking was not established.

For the period 2015 to 2025, there were *n* = 493 published systematic reviews and meta-analyses related to glaucoma care, excluding scoping and other types of qualitative or narrative reviews, in peer-reviewed journals. Eligible systematic reviews and meta-analyses were categorised into domains independently by one reviewer (MPP). Any uncertainties in classification were resolved subsequently through discussion and consensus of a second reviewer (GV or AAB). The main domains of these systematic reviews or systematic reviews that included a meta-analysis are given in Table 1 and the list of titles is provided in Supplementary File 2.

**Table 1:** Summary of published systematic reviews or systematic reviews and meta-analyses from the years 2015 to 2025 (including non-Cochrane reviews).

Domain	Number of systematic reviews or meta-analyses published (2015–2025)
Treatment-Surgery	123
Risk Factors	77
Genetics	61
Treatment-Medical	45
Treatment-Laser	25
Prevalence	23
Investigations-Not specified	18
Artificial intelligence (AI)	13
Treatment-Other	12
Research Methods	11
Access to Care	10
Incidence	10
Quality of Life	9
Treatment-IVI	8
Guidelines	7
Investigations-OCT	7
Prognostic Factors	7
Cost-Effectiveness	4
Investigations-IOP	4
Investigations-OCTA	3
Treatment	3
Treatment-Surgery-Paediatrics	2
Investigations-AS	1
Investigations-AS OCT	1
Investigations-Doppler	1
Investigations-Home Based	1
Investigations-IOL	1
Investigations-Other	1
Investigations-Paediatric	1
Investigations-RNFL	1
Investigations-VR VF	1
Quality and Safety	1
Treatment-Surgery-OCT	1
**Total number of reviews**	**493**

*AS* anterior segment, *IOL* intraocular lens, *IOP* intraocular pressure, *IVI* intra vitreal injection, *OCT* optical coherence tomography, *OCTA* optical coherence tomography angiography, *RNFL* retinal nerve fibre layer, *VF* visual fields, *VR* virtual reality.

**Fig. 2:**
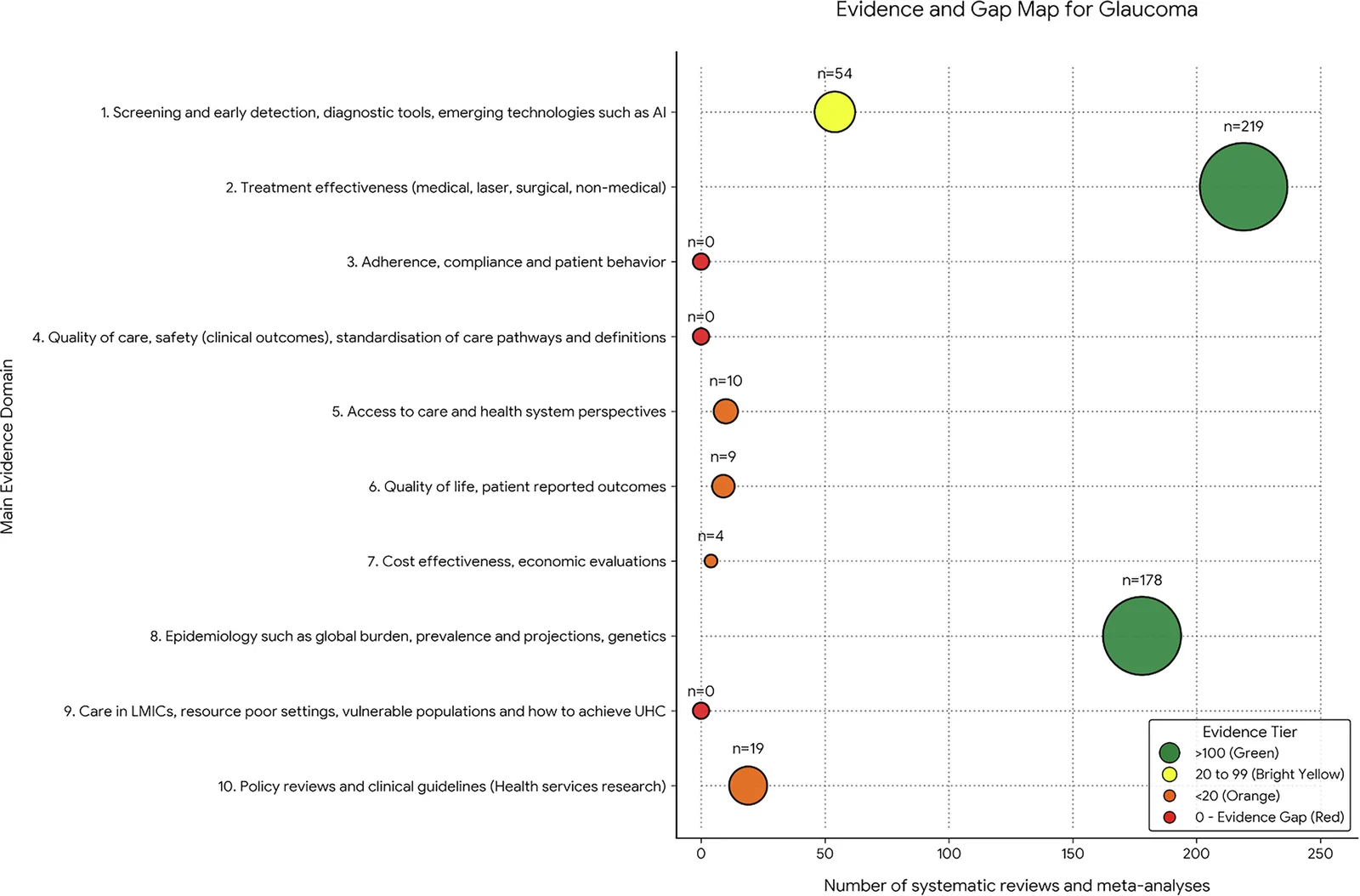
Summary of the Evidence and Gap Mapping outcomes. AI artificial intelligence, LMICs low- and middle-income countries, UHC universal health coverage.

A summary of the systematic reviews and meta-analyses conducted over the past decade indicates a predominant focus on identifying the risk factors (*n* = 77, 15.6%), genetic factors (*n* = 61, 12.4%) and effectiveness of various treatment options (*n* = 219, 44.4%) (see Fig. 2 for graphical presentation of the summary of EGM outcomes and Supplementary File 3 for a tree map showing absolute proportions of systematic reviews on each topic). However, in the absence of a coordinated, global level of framework to guide the identification of evidence gaps, there is a substantial possibility of duplication of research efforts by different evidence synthesis groups, unless this has been studied explicitly by the primary review groups themselves. Conversely, important gaps in the evidence base may remain unrecognised and unaddressed, unless the review groups considered a broader conceptual framework considering the full spectrum of epidemiology of glaucoma, glaucoma health promotion and service organisation. Based on our conceptual framework, one such critical gap pertains to the cost-effectiveness of screening and treatment options (0.8% of the published reviews for 2015–2025), which is essential for informing and optimising glaucoma eye care pathways and policy decisions. In our EGM, we also found that none of the included systematic reviews explored health system and policy-related questions, which is a major gap for global advocacy. Furthermore, none of the included systematic reviews examined gender-related disparities in access to glaucoma care, particularly in LMICs, highlighting an important gap in the evidence synthesis literature. This gap may also reflect a paucity of primary studies investigating gender-related disparities in access to glaucoma care.

## Reviews Published by the Cochrane Eyes and Vision Group in Glaucoma Care

Over the past decade, the CEV group has generated a substantial body of evidence addressing major gaps in evidence and informing clinical practice. During the period 2015 to 2025, the CEV group published 33 systematic reviews and meta-analyses in glaucoma care, providing a comprehensive overview of the available evidence spanning glaucoma screening, diagnosis, treatment and rehabilitation. The CEV glaucoma reviews are characterised by rigorous methodology by adhering to the Cochrane guidelines at each stage of the review process, including exhaustive literature searches, predefined eligibility criteria, structured risk of bias and study quality appraisal, grading of evidence and transparent reporting, ensuring a high level of credibility and reproducibility across the review process. The summary of outcomes or authors’ conclusions regarding Cochrane reviews for the last 10 years is given in Supplementary File 4. Table 2 provides a summary of the research areas/domains of the published Cochrane reviews.

**Table 2:** Main area of research and number of publications of Cochrane reviews for the period of 2015–2015.

Domain	Number of reviews	Author, year of the review
Diagnosis of glaucoma	*n* = 2	Michelessi [37], Jindal [11]
Surgical techniques	*n* = 16	Cabourne 2015, Zhnag 2015, Cheng 2016, Michelessi 2016, Tseng 2017, King 2018, Foo 2019, Le 2019, Otarola 2021, Ng 2021, Sandhu 2021, Theventhiran 2021, Ong 2021, Hu 2021, Park 2023, Gagrani 2020
Laser procedures	*n* = 8	Zhang 2017, Michelessi 2018, Ta 2019, Chen 2019, Bayliss 2021, Rolim-de-Moura 2022, Rouse 2023, Ong 2023
Topical Medication	*n* = 3	Xu 2017, Sena 2017, Clement 2022
Anti-VEGF	*n* = 1	Rittiphairoj 2023
Other treatment options such as acupuncture	*n* = 1	Law 2019
Behavioural intervention	*n* = 1	Kruoch 2023
Prognostic factors	*n* = 1	Piyasena [54]
**Total number of reviews**	**33**	

*Anti-VEGF* anti-vascular endothelial growth factor, *VEGF* vascular endothelial growth factor.

## Quality of Systematic Reviews Published

While systematic reviews and meta-analyses are widely regarded as the highest levels of evidence in clinical medicine, a growing body of empirical research shows that many published systematic reviews suffer from substantive methodological and reporting weaknesses. These shortcomings can undermine the reliability of their conclusions and mislead clinicians, guideline developers and policymakers. Several large evaluations using validated appraisal tools—such as AMSTAR-2 (A MeaSurement Tool to Assess Systematic Reviews) and ROBIS (Risk of Bias in Systematic Reviews)—have documented widespread quality issues across published reviews [18–20]. Structured methods, including a priori protocols, exhaustive search strategies, standardised bias assessments and rigorous editorial oversight [21], are designed to minimise these risks and have been shown repeatedly to produce higher methodological quality reviews.

Gao et al. [22] and Lee et al. [23], using AMSTAR-based evaluation, showed that most ophthalmology systematic reviews were of low or moderate quality, with major deficits in protocol registration and publication bias assessment. An overview of systematic reviews conducted by Qureshi et al. [24] on the topic of *‘What we really know about the effectiveness of glaucoma interventions’* highlighted that the majority of systematic reviews evaluating the effectiveness of interventions for glaucoma have poor reliability; even among those considered to be reliable there were uncertainties including the effectiveness of behavioural and educational interventions to improve adherence to glaucoma medication; comparative effectiveness of combined glaucoma and cataract surgery; comparative effectiveness of different laser treatment for treatment of primary angle-closure glaucoma; effectiveness of minimally invasive glaucoma surgery and effectiveness of glaucoma drainage device or aqueous shunts compared with trabeculectomy [24]. In that review, 129 eligible studies were evaluated, with 17 reviews of medical interventions for glaucoma, 16 reviews of surgical and post-operative interventions in glaucoma and 11 reviews of peri-operative interventions considered to be reliable, with only 38% of published systematic reviews considered reliable [24].

An analysis of Cochrane reviews and randomised trials conducted by Bondok et al. [25] reported that equity considerations should be addressed in research on glaucoma in terms of reporting socio-demographic characteristics of the study participants of RCTs, particularly race and ethnicity. Bondok et al. [25] highlighted that global representation of the participants included in glaucoma research was insufficient, especially those involving people from low-income countries, leading to concerns regarding generalisability of the findings [25]. On the other hand, in order to have high-quality systematic reviews, high-quality primary studies are needed. A systematic review by Higgins et al. assessing the reporting standards in glaucoma studies utilising electronic health records (EHRs), reported that of the 31 identified studies, only 16% adhered fully to the CODE-EHR best practice framework’s minimum standards, with none meeting the preferred standards [26]. Similarly, a systematic review by Leonard-Hawkhead et al. found that overall reporting of important aspects of AI studies was suboptimal in terms of adhering to standard reporting guidelines such as development and exploratory clinical investigations of decision support systems driven by artificial intelligence (DECIDE-AI) (underreporting of AI-specific items 30.3%) [27].

## Novel Strategies and the Way Forward

As early glaucoma is asymptomatic, a large proportion of cases remain undiagnosed [28]. Despite the intuitive appeal of screening, several barriers limit its viability. Current approaches to diagnose glaucoma rely on opportunistic screening, including cases identified during healthcare encounters (optometry, diabetic retinopathy programmes, cataract clinics, etc.) [29]. Population screening is not recommended in high-income countries due to low disease prevalence, imperfect tests and unfavourable economics [30]. As a key illustration: retinal nerve fibre layer (RNFL) OCT, with 79% sensitivity and 92% specificity, yields a positive predictive value of only ~23% at a 2.93% population prevalence, meaning that 77% of positive screens are false positives [31]. However, screening may be cost-effective in some LMIC settings (e.g., China, India), particularly where angle-closure glaucoma is more prevalent, and labour costs are lower [32]. Developing glaucoma detection within broader eye health screening programmes (e.g., diabetic retinopathy, age-related macular degeneration) is proposed as a pragmatic, sustainable alternative, supported by evidence from large US community studies [33] and the Beijing Eye Public Health Care Project [34]. Emerging technologies can offer potential to improve screening viability; for example, polygenic risk scores (PRS), built from 127+ genome-wide association studies that identified loci [35], where individuals in the top PRS decile may carry a 15-fold greater risk of advanced glaucoma than those in the bottom decile [36].

A meta-analysis by Michelessi et al. [37] reported that the accuracy of imaging for detecting manifest glaucoma was variable across different studies, and the current evidence base could be an overestimation. The authors of this review suggested that more evidence on DTA for detecting glaucoma using various imaging modalities was required [37]. A review by Fallon et al. [38] in assessing the effectiveness of Heidelberg retinal tomograph-3 (HRT-3), scanning laser polarimetry (GDx), and OCT also suggested that DTA in current studies could be an overestimation, although the screening studies showed lower sensitivity values [38]. A critical appraisal of the current evidence base highlights the need for more robust studies to evaluate the diagnostic accuracy and longitudinal performance of screening modalities for glaucoma detection and progression assessment.

## AI on Glaucoma Diagnosis, Detection and Assessment of Progression

Digital innovations in information and communications technology, including AI, are likely to broaden the scope for development of new strategies for better diagnosis of glaucoma, as well as detection of disease progression [39]. *Optic disc and cup analysis:* Deep learning models automatically segment the optic disc, calculate cup-to-disc ratios and detect structural features (e.g., rim thinning, RNFL defects, disc haemorrhage) from colour fundus photographs with accuracy meeting or exceeding that of trained clinicians [40, 41]. *OCT integration:* Spectral-domain OCT combined with AI enables quantification of peripapillary RNFL thickness, macular ganglion cell–inner plexiform layer (GCIPL) complex and optic nerve head morphology, supporting pre-perimetric glaucoma detection before visual field (VF) loss [41]. Novel segmentation architectures incorporating multiple view-specific modules (circumpapillary circle, macular cube and radial scans) enhance automated OCT analysis further [42]. *Multimodal data fusion:* AI systems integrating imaging with EHR data, intra ocular pressure and patient demographics improve diagnostic stratification and personalised risk assessment beyond imaging alone [42, 43].

### Colour Fundus Photography

Colour fundus photography is a widely accessible imaging modality that can be integrated with deep learning models to develop automated screening systems for glaucoma. Deep learning models analysing colour fundus photography achieved a pooled sensitivity of 0.92 (95% CI: 0.89–0.94) and specificity of 0.93 (95% CI: 0.90–0.95) (area under the receiver operator characteristic curve [AUC] = 0.90) in detecting glaucoma, based on a systematic review and meta-analysis of 48 studies [40]. Offline AI systems coupled with portable fundus cameras demonstrated high accuracy in real-world rural tele-ophthalmology settings, with a prospective study achieving sensitivity and specificity comparable to expert glaucoma specialists [44]. Smartphone-integrated AI systems may enable cost-effective, decentralised screening that can be readily deployed in low-resource settings. Multimodal pipelines combining image enhancement, automated optic disc/cup segmentation and deep learning classification achieved a sensitivity of 0.81 and specificity of 0.91 for early disc haemorrhage and RNFL defects in real-world validation [45]. Foundation models represent a rapidly evolving class of deep learning systems with broad potential application in glaucoma imaging. As an example, foundation models such as *RETFound*, trained on 1.6 million retinal images via self-supervised masked auto-encoders, outperform conventional ImageNet-pretrained networks, show resilience to poor imaging conditions and improve screening accessibility in resource-limited environments [42].

### Visual Field Forecasting From Macular OCT

Recent advances in AI have shown promise for predicting glaucoma progression using multimodal clinical data. Vision transformer models pretrained on 7.7 million macular OCT B-scans can detect concurrent VF progression with high accuracy (AUC 0.90) and predict future progression before it becomes clinically apparent, achieving an AUC of 0.86 for any progression, as well as up to 0.93 for rapid progression, defined as a deterioration in mean deviation exceeding 1 dB/year [46]. *Serial optic disc photograph analysis:* Longitudinal analysis of optic disc photographs (ODPs) has also demonstrated strong predictive performance. Siamese neural network models based on ResNet-152 architecture achieved an AUC of 0.81 using baseline ODPs alone, increasing to 0.86 with two serial ODPs and 0.89 with three serial images when combined with RNFL thickness measurements [47]. Similarly, a twin-network model based on ResNet-50 trained on longitudinal ODP pairs achieved an AUC of 0.86 for predicting any VF progression and 0.93 for rapid progression, suggesting that AI may facilitate earlier identification of patients requiring treatment escalation [47]. *EHR and natural language processing:* AI models incorporating EHR data have further expanded prognostic capabilities. Multimodal deep language models integrating free-text operative notes with structured EHR data achieved a macro AUROC of 0.75 for predicting glaucoma surgical outcomes, outperforming models based solely on structured clinical data (AUROC = 0.71) [42]. In addition, natural language processing applied to free-text clinical records has demonstrated potential for predicting short-term glaucoma progression and the need for surgical intervention [48].

Despite these encouraging findings, several challenges continue to limit the clinical implementation of AI in glaucoma care. These include heterogeneity of training datasets, which restricts model generalisability; inconsistent reporting and evaluation standards; a lack of prospective validation in real-world clinical settings; limited model interpretability associated with ‘*black-box*’ algorithms; regulatory uncertainty and concerns regarding health equity, as AI performance may vary across different ethnic populations and imaging platforms [48].

## Translating Evidence into Policy and Practice

Effective translation of evidence into glaucoma care policy and practice requires strategic partnership between evidence synthesis groups and global advocacy organisations. One example is a partnership between the American Academy of Ophthalmology, the European Glaucoma Society (EGS) and the CEV USA that has contributed to improving the clinical guidelines for glaucoma. An important contribution of the CEV USA group has been its critical appraisal of the reliability of published systematic reviews in glaucoma over the period from 2014 to 2024. The finding that reliability indices ranged from 21 to 38% highlights substantial variability in the methodological quality of existing reviews and underscores the risks of relying on inadequately conducted evidence syntheses for guideline development. Another key contribution of CEV through evidence synthesis projects has been to inform the development of the last two editions of the EGS Guidelines, which have included only landmark trials and high-quality systematic reviews to inform their content [49, 50].

There are also global indicators for assessing the screening and treatment coverage for major causes of blindness worldwide such as cataract surgical rate and effective refractive error coverage, as defined in the global advocacy documents under the WHO. However, such indicators are not currently provided for glaucoma based on global consensus and addressing this at a global advocacy level is a very high priority [51]. Liang et al. proposed three indicators for glaucoma, namely Glaucoma Coverage Rate, Glaucoma Detection Rate and Glaucoma Follow-up Adherence Rate, which are useful for monitoring the glaucoma eye care health system performance [52].

## Conclusion and Recommendations for Conducting Future Research/Systematic Reviews in Glaucoma Care

High-quality systematic reviews and meta-analyses provide the strongest evidence for clinical guideline development, as they synthesise all potentially relevant primary studies on glaucoma interventions by applying robust methodologies [53]. When evidence is incorporated into clinical practice guidelines, systematic reviews help standardisation of care, supporting clinicians to adopt evidence-based approaches that can reduce the variations in diagnosis and management of glaucoma across regions and healthcare systems in different country income settings. There should also be opportunities for adaptation of these interventions for local contextual requirements, improving the cultural relevance and acceptance by service users and service providers.

It was observed that the Cochrane reviews, as well as the majority of published reviews during the last 10 years, focused on the effectiveness of surgical interventions (16/33, 48%) and laser treatment procedures (8/33, 24%). However, substantial gaps remain in the Cochrane evidence synthesis, particularly regarding access to care, health system perspectives, cost-effectiveness of the interventions, care in LMICs and policy reviews related to improving glaucoma care. Overall, the existing evidence largely addresses the evidence gaps needed for day-to-day clinical practice, with more evidence required in areas such as prognostic factors [54] to inform efficient utilisation of available resources. Policy decisions that are guided by high-quality systematic reviews enable prioritisation of different strategies or interventions, ensuring that future clinical and public health policies continually improve detection strategies and therapeutic effectiveness in glaucoma care. Systematic reviews also highlight gaps and uncertainties in current glaucoma evidence, which provide the basis for targeted primary research studies, ensuring that future clinical practice guidelines and public health policies improve continually.

In this review, we observed that most of the Cochrane reviews synthesise relevant and important evidence, allowing readers to reach clinically applicable conclusions informing clinical practice guidelines and policy [55]. Some reviews highlight the current uncertainty and are unable to reach definite conclusions. In addition, existing systematic reviews on glaucoma care predominantly focus on risk factors, genetic influences and the effectiveness of treatments, especially surgical interventions, but there are significant gaps in several other critical areas that warrant further evidence synthesis and primary research. Future primary research and systematic reviews should prioritise exploring research questions such as the cost-effectiveness of screening and treatment strategies at a population level, the application of AI in diagnosis and treatment and efficiency in care pathways in terms of health systems perspectives, using ‘*real-world*’ data. Further research is also needed to evaluate the impact of current policy decisions on glaucoma care in terms of prevention of vision impairment and blindness, generating more evidence on paediatric glaucoma and exploring the effectiveness of interventions in LMICs. Achieving universal eye health coverage for glaucoma remains a key objective, requiring more primary research into equitable access to care and addressing gender disparities. By addressing these gaps, future primary and evidence synthesis studies can help optimise care, promote more inclusive glaucoma care policies and ensure that evidence-based practices are applied globally. Furthermore, it is imperative to improve the quality of systematic review outputs by adhering to standard guidelines in conducting and reporting evidence synthesis projects.

## Supplementary Information

Supplementary Information

## Data Availability

No datasets were generated or analysed during the current study.
