# Supplementary material for: Progress in the Synthesis of Evidence in Glaucoma: What We Know and the Gaps that Remain
Source: Ophthalmic Physiol Opt. 2026 Oct 19;46(6):1296–304. doi: 10.1007/s44402-026-00218-w (PMC13694298; doi:10.1007/s44402-026-00218-w)
Supplement: Supplementary file 1 [file 44402_2026_218_MOESM1_ESM.docx]

**SUPPLEMENTARY FILES**

**Supplementary File 1 - Electronic database search strategy**

(Glaucoma or Primary Open Angle).af.

(screening or detection or diagnosis).af.

(Treatment or eye drops or medical or surgical or laser or trabeculectomy).af.

(adherence or compliance or behaviour or barriers or Facilitators or enablers or Patient or Challenges).af.

(Quality of Care or safety or clinical outcomes).af.

(Access or health system or hospital or eye clinic or glaucoma clinic).af.

(Quality of Life or QALY or DALY).af.

(Cost effectiveness or ICER or OOP or WTP).af.

(Global Burden or Prevalence or Incidence or Projection or Rates).af.

(Vulnerable Population or Equity or Equality).af.

(LMIC or UMIC or LIC or Resource poor settings).af.

(Policy Review or Clinical Guidelines).af.

(Systematic Review or Meta-analysis or Meta analysis).af.

**Supplementary File 2 - Table 1. Titles of all reviews included in the Evidence and Gap Mapping exercise (2015 to 2025)**

| **Category** | **Title of the Review** |
| --- | --- |
| Access to Care | Adherence and Persistence on Prostaglandin Analogues for Glaucoma: A Systematic Review and Meta-Analysis. |
| Access to Care | Interventions for Glaucoma Medication Adherence Improvement: A Network Meta-analysis of Randomized Controlled Trials. [Review] |
| Access to Care | Aiding Adherence to Glaucoma Medications: A Systematic Review. |
| Access to Care | Comparative effectiveness of interventions for improving adherence to ocular hypotensive therapy in patients with glaucoma or ocular hypertension: protocol for network meta-analysis. |
| Access to Care | Systematic Review of Knowledge Assessments for Glaucoma Patients. |
| Access to Care | Topical medication instillation techniques for glaucoma. |
| Access to Care | Barriers and enablers to medication adherence in glaucoma: A systematic review of modifiable factors using the Theoretical Domains Framework. |
| Access to Care | Adherence and factors influencing adherence to glaucoma medications among adult glaucoma patients in Ethiopia: A systematic review and meta-analysis. |
| Access to Care | Public awareness about glaucoma, cataract, and diabetic retinopathy in Saudi Arabia: a systematic review and meta-analysis. |
| Access to Care | Evaluating Measurement Properties of Patient-Reported Outcome Measures in Glaucoma: A Systematic Review. |
| AI | Artificial intelligence in the prediction of glaucoma development and progression: A systematic review. |
| AI | AI for glaucoma, Are we reporting well? a systematic literature review of DECIDE-AI checklist adherence. |
| AI | Artificial Intelligence in Anterior Chamber Evaluation: A Systematic Review and Meta-Analysis. |
| AI | Artificial intelligence in glaucoma: opportunities, challenges, and future directions. |
| AI | The role of artificial intelligence in analysis of biofluid markers for diagnosis and management of glaucoma: A systematic review. |
| AI | Diagnostic Accuracy of Artificial Intelligence in Glaucoma Screening and Clinical Practice. |
| AI | Performances of Machine Learning in Detecting Glaucoma Using Fundus and Retinal Optical Coherence Tomography Images: A Meta-Analysis. [Review] |
| AI | Accuracy of Using Generative Adversarial Networks for Glaucoma Detection: Systematic Review and Bibliometric Analysis. |
| AI | Deep learning versus ophthalmologists for screening for glaucoma on fundus examination: A systematic review and meta-analysis. |
| AI | Applications of deep learning in detection of glaucoma: A systematic review. |
| AI | Machine learning applied to retinal image processing for glaucoma detection: review and perspective. [Review] |
| AI | Deep Learning for Accurate Diagnosis of Glaucomatous Optic Neuropathy Using Digital Fundus Image: A Meta-Analysis. |
| AI | Health care cost and benefits of artificial intelligence-assisted population-based glaucoma screening for the elderly in remote areas of China: a cost-offset analysis. |
| Cost Effectiveness | Systematic methodological review of health state values in glaucoma cost-utility analyses. |
| Cost Effectiveness | A Review of Cost-Effectiveness Analyses for Open Angle Glaucoma Management. |
| Cost Effectiveness | Cost-effectiveness and cost-utility of population-based glaucoma screening in China: a decision-analytic Markov model. |
| Cost Effectiveness | Cost-effectiveness analysis of standalone trabecular micro-bypass stents in patients with mild-to-moderate open-angle glaucoma in Canada. |
| Genetics | A systematic review, meta-analysis, and network analysis of diagnostic microRNAs in glaucoma. |
| Genetics | Phenotypic and Genetic Links between Body Fat Measurements and Primary Open-Angle Glaucoma. |
| Genetics | The Association of Female Reproductive Factors with Glaucoma and Related Traits: A Systematic Review. |
| Genetics | Genetic Epidemiology of Primary Congenital Glaucoma in the 22 Arab Countries: A Systematic Review. |
| Genetics | Polymorphisms in CYP1B1 gene and the risk of suffering Primary Open-Angle Glaucoma: Systematic review and meta-analysis. |
| Genetics | Is Genetic Risk for Sleep Apnea Causally Linked With Glaucoma Susceptibility?. [Review] |
| Genetics | Genome-Wide Association Study Identifies Two Common Loci Associated with Pigment Dispersion Syndrome/Pigmentary Glaucoma and Implicates Myopia in its Development. |
| Genetics | Penetrance of MYOC gene mutation in primary open-angle glaucoma: A systematic review and meta-analysis. |
| Genetics | Meta-analysis of CYP1B1 gene mutations in primary congenital glaucoma patients. [Review] |
| Genetics | LOXL1 gene polymorphisms are associated with exfoliation syndrome/exfoliation glaucoma risk: An updated meta-analysis. |
| Genetics | Genome-wide meta-analysis identifies 127 open-angle glaucoma loci with consistent effect across ancestries. |
| Genetics | The role of polymorphisms rs2070744 and rs1799983 eNOS gene in patients with POAG: a systematic review and meta-analysis. |
| Genetics | Genetic association between CDKN2B/CDKN2B-AS1 gene polymorphisms with primary glaucoma in a North Indian cohort: an original study and an updated meta-analysis. |
| Genetics | Association of interleukin-6 gene polymorphisms and glaucoma: Systematic review and meta-analysis. |
| Genetics | Association of the CAV1-CAV2 locus with normal-tension glaucoma in Chinese and Japanese. |
| Genetics | Plasma lipid levels and risk of primary open angle glaucoma: a genetic study using Mendelian randomization. |
| Genetics | Vitamin D and its receptor polymorphisms are associated with glaucoma. |
| Genetics | Association of Glaucoma Risk Genes with Retinal Nerve Fiber Layer in a Multi-ethnic Asian Population: The Singapore Epidemiology of Eye Diseases Study. |
| Genetics | Comprehensive bioinformatics analysis of trabecular meshwork gene expression data to unravel the molecular pathogenesis of primary open-angle glaucoma. |
| Genetics | Genome-wide association analysis of 95 549 individuals identifies novel loci and genes influencing optic disc morphology. |
| Genetics | Multi-trait genome-wide association study identifies new loci associated with optic disc parameters. |
| Genetics | Associations between TLR4 Polymorphisms and Open Angle Glaucoma: A Meta-Analysis. |
| Genetics | Association of MTHFR C677T and A1298C Polymorphisms with Glaucoma Risk: a Systematic Review Meta-Analysis based 42 Case-Control Studies. |
| Genetics | Association of Polymorphisms at the SIX1-SIX6 Locus With Primary Open-Angle Glaucoma. |
| Genetics | Heritability of glaucoma and glaucoma-related endophenotypes: Systematic review and meta-analysis. |
| Genetics | Association of Gene Polymorphisms With Primary Open Angle Glaucoma: A Systematic Review and Meta-Analysis. |
| Genetics | The association of toll-like receptor 4 gene polymorphisms with primary open angle glaucoma susceptibility: a meta-analysis. |
| Genetics | Correlation between MTHFR polymorphisms and glaucoma: A meta-analysis. |
| Genetics | Genome-wide analyses identify 68 new loci associated with intraocular pressure and improve risk prediction for primary open-angle glaucoma. |
| Genetics | Cross-ancestry genome-wide association analysis of corneal thickness strengthens link between complex and Mendelian eye diseases. |
| Genetics | Heritability of glaucoma and glaucoma-related endophenotypes: systematic review and meta-analysis protocol. |
| Genetics | New insights into the genetics of primary open-angle glaucoma based on meta-analyses of intraocular pressure and optic disc characteristics. |
| Genetics | Association of WDR36 polymorphisms with primary open angle glaucoma: A systematic review and meta-analysis. |
| Genetics | Genetic analysis of Japanese primary open-angle glaucoma patients and clinical characterization of risk alleles near CDKN2B-AS1, SIX6 and GAS7. |
| Genetics | Association of Interleukin-1 gene clusters polymorphisms with primary open-angle glaucoma: a meta-analysis. |
| Genetics | Associations between matrix metalloproteinase gene polymorphisms and glaucoma susceptibility: a meta-analysis. [Review] |
| Genetics | CDKN2B gene rs1063192 polymorphism decreases the risk of glaucoma. |
| Genetics | Genetic Associations of Primary Angle-Closure Disease: A Systematic Review and Meta-analysis. |
| Genetics | Genome-wide association study identifies five new susceptibility loci for primary angle closure glaucoma. |
| Genetics | Genome-wide association analysis identifies TXNRD2, ATXN2 and FOXC1 as susceptibility loci for primary open-angle glaucoma. |
| Genetics | Association between the p53 codon 72 polymorphism and primary open-angle glaucoma risk: Meta-analysis based on 11 case-control studies. [Review] |
| Genetics | Association of MMP-9 Gene Polymorphisms with Glaucoma: A Meta-Analysis. [Review] |
| Genetics | LOXL1 Gene Polymorphism With Exfoliation Syndrome/Exfoliation Glaucoma: A Meta-Analysis. |
| Genetics | Genetic associations in PLEKHA7 and COL11A1 with primary angle closure glaucoma: a meta-analysis. |
| Genetics | Association of single nucleotide polymorphisms in the CYP1B1 gene with the risk of primary open-angle glaucoma: a meta-analysis. [Review] |
| Genetics | Ethnicity-Based Differences in the Association of LOXL1 Polymorphisms with Pseudoexfoliation/Pseudoexfoliative Glaucoma: A Meta-Analysis. |
| Genetics | Associations of polymorphisms of LOXL1 gene with primary open-angle glaucoma: a meta-analysis based on 5,293 subjects. |
| Genetics | The effect of MTHFR ala222val polymorphism on open-angle glaucoma: a meta-analysis. |
| Genetics | Genetic Associations of Primary Angle-Closure Disease: A Systematic Review and Meta-Analysis. |
| Genetics | Association of Gene Polymorphisms with Normal Tension Glaucoma: A Systematic Review and Meta-Analysis. |
| Genetics | Consolidating data on the association of IL-6 and IL-10 polymorphisms with the development of glaucoma: a meta-analysis. |
| Genetics | Lack of Association between LOXL1 Variants and Pigment Dispersion Syndrome/Pigmentary Glaucoma: A Meta-Analysis. |
| Genetics | Genetic Basis of Pigment Dispersion Syndrome and Pigmentary Glaucoma: An Update and Functional Insights. [Review] |
| Genetics | Genetic changes and testing associated with childhood glaucoma: A systematic review. |
| Genetics | Genetic association of IL1B gene variants with primary glaucoma in a North Indian Punjabi cohort: An original study and meta-analysis. |
| Genetics | Association between optic atrophy 1 polymorphisms and primary open angle glaucoma risk: Based on a meta-analysis. |
| Genetics | Effect of genotype on individual response to the pharmacological treatment of glaucoma: a systematic review and meta-analysis. |
| Genetics | Genetic associations in CHAT and COL11A1 with primary angle-closure glaucoma susceptibility: A systematic review and meta-analysis. |
| Genetics | Glaucoma Genetic Risk Scores in the Million Veteran Program. |
| Genetics | Genetic association between CDKN2B-AS1 polymorphisms and the susceptibility of primary open-angle glaucoma (POAG): a meta-analysis from 21,775 subjects. |
| Genetics | Meta-analysis of Genome-Wide Association Studies Identifies Novel Loci Associated With Optic Disc Morphology. |
| Guidelines | Network Meta-analysis for Clinical Practice Guidelines: A Case Study on First-Line Medical Therapies for Primary Open-Angle Glaucoma. |
| Guidelines | Guidelines for treatment of chronic primary angle-closure glaucoma. |
| Guidelines | Screening for Primary Open-Angle Glaucoma: US Preventive Services Task Force Recommendation Statement. |
| Guidelines | Screening for Glaucoma in Adults: Updated Evidence Report and Systematic Review for the US Preventive Services Task Force. |
| Guidelines | Evidence-based practice guideline of Chinese herbal medicine for primary open-angle glaucoma (qingfeng -neizhang). |
| Guidelines | Assessing the Methodological Quality of Glaucoma Clinical Practice Guidelines and Their Recommendations on Microinvasive Glaucoma Surgery: A Systematic Review. |
| Guidelines-Paed | A systematic review of clinical practice guidelines for childhood glaucoma. |
| Incidence | Incidence and Association of Angle Closure Glaucoma in Retinitis Pigmentosa: A Meta-Analysis. |
| Incidence | Incidence of Glaucoma in Type 2 Diabetes Patients Treated With GLP-1 Receptor Agonists: A Systematic Review and Meta-Analysis. [Review] |
| Incidence | The Incidence and Presentation Features of Glaucoma in Vogt-Koyanagi-Harada Syndrome: A Systematic Review and Meta-Analysis. |
| Incidence | Global incidence and risk factors for glaucoma: A systematic review and meta-analysis of prospective studies. |
| Incidence | Incidence Rate of Secondary Glaucoma Following Congenital Cataract Surgery: An In-Depth Systematic Review and Meta-Analysis. |
| Incidence | The Association Between Eye Disease and Incidence of Dementia: Systematic Review and Meta-Analysis. |
| Incidence | The incidence and risk factors of neovascular glaucoma secondary to proliferative diabetic retinopathy after vitrectomy. |
| Incidence | GLP-1 Receptor Agonists Use and Incidence of Glaucoma: A Systematic Review and Meta-Analysis. |
| Incidence | OCULAR HYPERTENSION AND GLAUCOMA FOLLOWING VITRECTOMY: A Systematic Review. |
| Incidence-Paed | The role of primary intraocular lens implantation in the risk of secondary glaucoma following congenital cataract surgery: A systematic review and meta-analysis. |
| Investigations-OCTA | Optic nerve head vessel density using OCTA in patients with primary angle closure disease: A systematic review and network meta-analysis. |
| Investigations | Virtual reality perimetry compared to standard automated perimetry in adults with glaucoma: A systematic review. |
| Investigations | Assessing the value of the water drinking test in glaucoma-a systematic review and meta-analysis. |
| Investigations | Platelet-to-lymphocyte ratio and lymphocyte-to-monocyte ratio in glaucoma: a meta-analysis. [Review] |
| Investigations | Optic nerve head vessel density in patients with pseudoexfoliation syndrome/glaucoma: A systematic review and meta-analysis. |
| Investigations | Non-contact tests for identifying people at risk of primary angle closure glaucoma. |
| Investigations | Glaucoma due to ciliary body cysts and pseudoplateau iris: a systematic review of the literature. |
| Investigations | Corneal properties and glaucoma: a review of the literature and meta-analysis. [Review] |
| Investigations | Diagnostic accuracy of imaging devices in glaucoma: A meta-analysis. [Review] |
| Investigations | Oxidative and Anti-Oxidative Stress Markers in Chronic Glaucoma: A Systematic Review and Meta-Analysis. |
| Investigations | Choroidal Thickness and Open-Angle Glaucoma: A Meta-Analysis and Systematic Review. |
| Investigations | Literature review and meta-analysis of translaminar pressure difference in open-angle glaucoma. |
| Investigations | Confocal scan laser ophthalmoscope for diagnosing glaucoma: a systematic review and meta-analysis. |
| Investigations | Aqueous humor TGF-beta2 levels in patients with open-angle glaucoma: A meta-analysis. |
| Investigations | Corneal Biomechanics Assessment with Ultra High Speed Scheimpflug Camera in Primary Open Angle Glaucoma Compared with Healthy Subjects: A meta-analysis of the Literature. |
| Investigations | Diagnostic performance of laser speckle flowgraphy in glaucoma: a systematic review and meta-analysis. |
| Investigations | Electrophysiology in Glaucoma. [Review] |
| Investigations | Validity and reliability of eye healthcare professionals in the assessment of glaucoma - a systematic review. |
| Investigations-AS | Anterior segment imaging in minimally invasive glaucoma surgery - A systematic review. |
| Investigations-AS OCT | Diagnostic accuracy of AS-OCT vs gonioscopy for detecting angle closure: a systematic review and meta-analysis. |
| Investigations-Doppler | Colour Doppler analysis of ophthalmic vessels in the diagnosis of carotic artery and retinal vein occlusion, diabetic retinopathy and glaucoma: systematic review of test accuracy studies. |
| Investigations-Home Based | Home-Based Perimetry for Glaucoma: Where Are We Now?. |
| Investigations-IOL | A systemic review and network meta-analysis of accuracy of intraocular lens power calculation formulas in primary angle-closure conditions. |
| Investigations-IOP | Agreement and Reliability of Transpalpebral Tonometers with Goldmann Applanation Tonometer: A Systematic Review and Meta-analysis. |
| Investigations-IOP | Evaluation of the agreement and reliability of Transpalpebral Tonometers compared with Goldmann Applanation Tonometer - A systematic Review and Meta-Analysis Protocol. |
| Investigations-IOP | Meta-analysis of the concordance of Icare R PRO-based rebound and Goldmann applanation tonometry in glaucoma patients. |
| Investigations-IOP | Systematic Review of Current Devices for 24-h Intraocular Pressure Monitoring. |
| Investigations-OCT | Age-related changes in optical coherence tomography glaucoma-related parameters: A systematic review. |
| Investigations-OCT | Peripapillary Retinoschisis in Glaucoma: A Systematic Review. |
| Investigations-OCT | The Diagnostic Value of Optical Coherence Tomography for Detecting Glaucoma in High Myopia. |
| Investigations-OCT | A system review and meta-analysis of the treatment of acute angle-closure glaucoma under optical coherence tomography. |
| Investigations-OCT | Accuracy of optical coherence tomography for diagnosing glaucoma: an overview of systematic reviews. |
| Investigations-OCT | Microvascular damage assessed by optical coherence tomography angiography for glaucoma diagnosis: a systematic review of the most discriminative regions. |
| Investigations-OCT | Optical coherence tomography for glaucoma diagnosis: An evidence based meta-analysis. |
| Investigations-OCTA | OCT-angiography detects longitudinal microvascular changes in glaucoma: a systematic review. |
| Investigations-OCTA | Diagnostic performance of optical coherence tomography angiography in glaucoma: a systematic review and meta-analysis. |
| Investigations-Other | Diagnostic accuracy of isolated-check visual evoked potentials for glaucoma: a systematic review and meta-analysis. |
| Investigations-Paed | Virtual Reality With Eye Tracking for Pediatric Ophthalmology: A Systematic Review. |
| Investigations-RNFL | Macular versus Retinal Nerve Fiber Layer Parameters for Diagnosing Manifest Glaucoma: A Systematic Review of Diagnostic Accuracy Studies. |
| Investigations-VR VF | The use of augmented reality and virtual reality for visual field expansion and visual acuity improvement in low vision rehabilitation: a systematic review. |
| Investigations | Comparison of Peripapillary Choroidal Thickness Between Primary Open-angle Glaucoma, Normal Tension Glaucoma, and Normal Eyes: A Systematic Review and Meta-analysis. |
| Prevalence | Prevalence of glaucoma in Africa: A systematic review and Bayesian meta-analysis. |
| Prevalence | Prevalence of MYOC risk variants for glaucoma in different populations. |
| Prevalence | Re: Tham et al.: Global prevalence of glaucoma and projections of glaucoma burden through 2040: a systematic review and meta-analysis (Ophthalmology 2014;121:2081-90). |
| Prevalence | National, regional, and provincial prevalence of glaucoma in China in 2020: an updated systematic review and modelling analysis. |
| Prevalence | Prevalence of Glaucoma in Europe and Projections to 2050: Findings from the European Eye Epidemiology Consortium. |
| Prevalence | Prevalence of Depression and Anxiety Disorders in Patients with Glaucoma: A Systematic Review and Meta-Analysis Based on Cross-Sectional Surveys. |
| Prevalence | Global estimates on the number of people blind or visually impaired by glaucoma: A meta-analysis from 2000 to 2020. |
| Prevalence | Prevalence Ratio of Primary Angle-Closure and Primary Open-Angle Glaucoma in Asian Population: A Meta-Analysis and Multiple Meta-Regression Analysis. |
| Prevalence | The prevalence and presentation patterns of microcystic macular oedema: a systematic review and meta-analysis of 2128 glaucomatous eyes. |
| Prevalence | Primary Open Angle Glaucoma Prevalence in Europe: A Systematic Review and Meta-Analysis. |
| Prevalence | Prevalence and diagnostic ability of beta-zone parapapillary atrophy in open-angle glaucoma: a systematic review and meta-analysis. |
| Prevalence | Prevalence of complications in eyes with nanophthalmos or microphthalmos: protocol for a systematic review and meta-analysis. |
| Prevalence | Myopic Open-angle Glaucoma Prevalence in Northeast Asia: A Systematic Review and Meta-analysis of Population-based Studies. |
| Prevalence | Blindness in glaucoma: primary open-angle glaucoma versus primary angle-closure glaucoma-a meta-analysis. |
| Prevalence | Prevalence of primary open angle glaucoma in the last 20 years: a meta-analysis and systematic review. |
| Prevalence | The Global Extent of Undetected Glaucoma in Adults: A Systematic Review and Meta-analysis. |
| Prevalence | Prevalence of Charles Bonnet syndrome in patients with glaucoma: a systematic review with meta-analyses. |
| Prevalence | Prevalence of Normal-Tension Glaucoma in the Chinese Population: A Systematic Review and Meta-Analysis. |
| Prevalence | National and subnational prevalence and burden of glaucoma in China: A systematic analysis. |
| Prevalence | Global variations and time trends in the prevalence of primary open angle glaucoma (POAG): a systematic review and meta-analysis. |
| Prevalence | Glaucoma in Asia: regional prevalence variations and future projections. |
| Prevalence | Number of People Blind or Visually Impaired by Glaucoma Worldwide and in World Regions 1990 - 2010: A Meta-Analysis. |
| Prevalence-IOP-Paed | Normal intraocular pressure in Egyptian children and meta-analysis. |
| Prognostic Factors | Prognostic factors associated with progression of open-angle glaucoma in adults. |
| Prognostic Factors | Prognostic factors associated with progression of open-angle glaucoma in adults. [Review] |
| Prognostic Factors | Progression of primary angle closure suspects: a systematic review and meta-analysis. |
| Prognostic Factors | Changes in individuals' glaucoma progression velocity after IOP-lowering therapy: A systematic review. |
| Prognostic Factors | Rates of visual field change and functional progression in glaucoma following trabecular microbypass implantation of iStent technologies: a meta-analysis. [Review] |
| Prognostic Factors | The Relevance of Arterial Blood Pressure in the Management of Glaucoma Progression: A Systematic Review. |
| Prognostic Factors | Association of Optic Disc Tilt and Torsion with Open-Angle Glaucoma Progression Risk: Meta-Analysis and Meta-Regression Analysis. [Review] |
| QoL | Systematic Review of Instruments for the Assessment of Patient-Reported Outcomes and Quality of Life in Patients with Childhood Glaucoma. |
| QoL | Anxiety, depression, and sleep-related outcomes of glaucoma patients: systematic review and meta-analysis. |
| QoL | How do different lighting conditions affect the vision and quality of life of people with glaucoma? A systematic review. |
| QoL | The impact of mild, moderate, and severe visual field loss in glaucoma on patients' quality of life measured via the Glaucoma Quality of Life-15 Questionnaire: A meta-analysis. |
| QoL | A Systematic Review of End-of-Life Visual Impairment in Open-Angle Glaucoma: An Epidemiological Autopsy. |
| QoL | Preference-based Glaucoma-specific Health-related Quality of Life Instrument: Development of the Health Utility for Glaucoma. |
| QoL-Driving | The Effect of Glaucomatous Visual Field Defects on Driving: A Systematic Review. |
| QoL-Fractures | Glaucoma and Risk of Fractures: A Systematic Review, Meta-Analysis, and Network Meta-Analysis. |
| QoL-Paed | Quality of life and caregiver burden in pediatric glaucoma: A systematic review. |
| Quality n Safety | Quality of Reporting Electronic Health Record Data in Glaucoma: A Systematic Literature Review. |
| Research Methods | Systematic review with cross sectional comparative analysis identifies adverse event reporting discrepancies between ClinicalTrials.gov and published glaucoma randomized controlled trials. |
| Research Methods | A Systematic Review of Sex-Based Reporting in Topical Glaucoma Medication Clinical Trials. |
| Research Methods | Inequities in glaucoma research: an analysis of Cochrane systematic reviews and randomized trials. |
| Research Methods | Systematic review of the method and quality of reporting of complications from studies evaluating innovative glaucoma surgical procedures. |
| Research Methods | Racial and Ethnic Disparities in Primary Open-Angle Glaucoma Clinical Trials: A Systematic Review and Meta-analysis. |
| Research Methods | Methodology and reporting of diagnostic accuracy studies of automated perimetry in glaucoma: evaluation using a standardised approach. |
| Research Methods | Outcome Measures in Glaucoma: A Systematic Review of Cochrane Reviews and Protocols. |
| Research Methods | Systematic review and appraisal of quality, definitions and treatment recommendations of clinical guidelines for glaucoma suspects. |
| Research Methods | A Systematic Review of Glaucoma Diagnosis in Prevalence Studies and Quality of Reporting. |
| Research Methods | Reporting Complications in Glaucoma Surgery: A Systematic Review. |
| Research Methods | A systematic review and meta-analysis of randomised controlled trials in the management of neovascular glaucoma: absence of consensus and variability in practice. |
| Risk Factors | Exploring the relationship between accommodation and intraocular pressure: a systematic literature review and meta-analysis. |
| Risk Factors | Identifying potential key metabolic pathways and biomarkers in glaucoma: a systematic review and meta-analysis. |
| Risk Factors | Risk of glaucoma to subsequent dementia or cognitive impairment: a systematic review and meta-analysis. |
| Risk Factors | Is Glaucoma a Two-Pressure-Related Optic Neuropathy? A Systematic Review and Meta-Analysis. |
| Risk Factors | Risk of Parkinson's disease in glaucoma patients: a systematic review and meta-analysis. |
| Risk Factors | Alcohol, Intraocular Pressure, and Open-Angle Glaucoma: A Systematic Review and Meta-analysis. |
| Risk Factors | The Correlation Between Body Weight and Intraocular Pressure. |
| Risk Factors | Obesity and risk of age-related eye diseases: a systematic review of prospective population-based studies. |
| Risk Factors | Changes in Intraocular Pressure During Hemodialysis: A Meta-analysis. |
| Risk Factors | Association of glaucoma with risk of retinal vein occlusion: A meta-analysis. [Review] |
| Risk Factors | Hyperlipidemia, Blood Lipid Level, and the Risk of Glaucoma: A Meta-Analysis. |
| Risk Factors | The impact of disc hemorrhage studies on our understanding of glaucoma: a systematic review 50 years after the rediscovery of disc hemorrhage. |
| Risk Factors | Migraine as a risk factor for primary open angle glaucoma: A systematic review and meta-analysis. |
| Risk Factors | Pathophysiology, diagnosis, and management of glaucoma associated with Sturge-Weber syndrome. |
| Risk Factors | Hypothyroidism as a risk factor for open angle glaucoma: A systematic review and meta-analysis. |
| Risk Factors | Lack of Association between Serum Vitamin B6, Vitamin B12, and Vitamin D Levels with Different Types of Glaucoma: A Systematic Review and Meta-Analysis. |
| Risk Factors | The association between cigarette smoking and primary open-angle glaucoma: a systematic review. |
| Risk Factors | Association of Matrix Metalloproteinase-9 (MMP9) Variants with Primary Angle Closure and Primary Angle Closure Glaucoma. |
| Risk Factors | Meta-Analysis of Association of Obstructive Sleep Apnea With Glaucoma. |
| Risk Factors | Intranasal corticosteroids do not affect intraocular pressure or lens opacity: a systematic review of controlled trials. |
| Risk Factors | A systematic review of ionizing radiation-induced glaucoma: clinical manifestations, pathogenesis, and current treatment approaches. |
| Risk Factors | The effect of digital devices screen use on intraocular pressure: A systematic review and meta-analysis. |
| Risk Factors | Risk factors for glaucoma in nanophthalmos - a systematic review and meta-analysis. |
| Risk Factors | Ocular and Systemic Risk Factors and Biomarkers for Primary Glaucoma: An Umbrella Review of Systematic Reviews With Meta-Analyses. |
| Risk Factors | The pivotal role of inflammatory factors in glaucoma: a systematic review. |
| Risk Factors | Relationship Between Chemokine in Aqueous Humor and Primary Glaucoma: A Meta-Analysis. |
| Risk Factors | The Multifaceted Nongenetic Risk Factors for Primary Open-angle Glaucoma: An Overview of Systematic Reviews. |
| Risk Factors | Associations of different types of statins with the risk of open-angle glaucoma: a systematic review and network meta-analysis. |
| Risk Factors | Comprehensive assessment of glaucoma in patients with high myopia: a systematic review and meta-analysis with a discussion of structural and functional imaging modalities. |
| Risk Factors | Impact of positive airway pressure therapy on intraocular pressure in obstructive sleep apnea: A systematic review. |
| Risk Factors | Endothelin-1 Plasma and Aqueous Humor Levels in Different Types of Glaucoma: A Systematic Review and Meta-Analysis. |
| Risk Factors | Association between glaucoma and the risk of Alzheimer's disease: A meta-analysis. |
| Risk Factors | Systematic Review and Meta-Analysis on the Association Between Daily Niacin Intake and Glaucoma. |
| Risk Factors | Ocular manifestations of obstructive sleep apnea: a systematic review and meta-analysis. |
| Risk Factors | Association between metformin use and the risk of developing open-angle glaucoma among patients with diabetes: a retrospective cohort study and meta-analysis. |
| Risk Factors | Changes in corneal biomechanics in patients with glaucoma: a systematic review and meta-analysis. |
| Risk Factors | Glial Cell Activation and Immune Responses in Glaucoma: A Systematic Review of Human Postmortem Studies of the Retina and Optic Nerve. |
| Risk Factors | Risk Factors for Primary Angle-closure Glaucoma: A Systematic Review and Meta-analysis of 45 Studies. |
| Risk factors | Relative Risks for Dementia among Individuals with Glaucoma: A Meta-Analysis of Observational Cohort Studies. |
| Risk Factors | Association between helicobacter pylori infection and primary open-angle glaucoma: a systematic review and meta-analysis. |
| Risk Factors | Hypertension as a Risk Factor for Primary Open-angle Glaucoma: A Meta-analysis. |
| Risk Factors | Obstructive sleep apnoea and glaucoma: a systematic review and meta-analysis. |
| Risk Factors | A Systematic Review and Meta-analysis of Systemic Antihypertensive Medications With Intraocular Pressure and Glaucoma. |
| Risk Factors | Cataract surgery and IOP: a systematic review of randomised controlled trials. |
| Risk Factors | Association of diabetes, fasting glucose, and the risk of glaucoma: a systematic review and meta-analysis. |
| Risk Factors | Corneal Biomechanics in Primary Open Angle Glaucoma and Ocular Hypertension: A Systematic Review and Meta-analysis. |
| Risk Factors | Rho kinase inhibitor for primary open-angle glaucoma and ocular hypertension. |
| Risk Factors | Topiramate-induced acute angle closure: A systematic review of case reports and case series. |
| Risk Factors | Diet, Oxidative Stress, and Blood Serum Nutrients in Various Types of Glaucoma: A Systematic Review. |
| Risk Factors | Meta-Analysis of Dyslipidemia and Blood Lipid Parameters on the Risk of Primary Open-Angle Glaucoma. |
| Risk Factors | Relationship between Diabetic Retinopathy and Primary Open-Angle Glaucoma: A Systematic Review and Meta-Analysis. |
| Risk Factors | Vitamin intake and glaucoma risk: A systematic review and meta-analysis. |
| Risk Factors | Circulating fatty acids and risk of primary open-angle glaucoma: A mendelian randomization study. |
| Risk Factors | Degree of Myopia and Glaucoma Risk: A Dose-Response Meta-analysis. [Review] |
| Risk Factors | Cholesterol and glaucoma: a systematic review and meta-analysis. |
| Risk Factors | Nonocular Influencing Factors for Primary Glaucoma: An Umbrella Review of Meta-Analysis. |
| Risk Factors | Effects of isometric resistance exercise of the lower limbs on intraocular pressure and ocular perfusion pressure among healthy adults: A meta-analysis. [Review] |
| Risk Factors | Personality types of patients with glaucoma: A systematic review of observational studies. |
| Risk Factors | Risk Factors for the Development of Ocular Hypertension After Keratoplasty: A Systematic Review. |
| Risk Factors | Risk of intraocular pressure elevation after topical steroids in children and adults: A systematic review. |
| Risk Factors | The effects of intravitreal injections on intraocular pressure and retinal nerve fiber layer: a systematic review and meta-analysis. |
| Risk Factors | A meta-analysis of the rate and related factors of glaucomatous optic nerve damage in patients with Glaucomatocyclitic Crisis. |
| Risk Factors | Ocular Perfusion Pressure and the Risk of Open-Angle Glaucoma: Systematic Review and Meta-analysis. |
| Risk Factors | Association between glaucoma and the risk of Alzheimer's disease: A systematic review of observational studies. |
| Risk Factors | The relation between dietary intake and glaucoma: a systematic review. |
| Risk Factors | The risk of glaucoma and serotonergic antidepressants: A systematic review and meta-analysis. |
| Risk Factors | Modifiable factors in the management of glaucoma: a systematic review of current evidence. |
| Risk Factors | Triglycerides and Open Angle Glaucoma - A Meta-analysis with meta-regression. |
| Risk Factors | The interrelation between hypothyroidism and glaucoma: a critical review and meta-analyses. [Review] |
| Risk Factors | The Association between Systemic Glucocorticoid Use and the Risk of Cataract and Glaucoma in Patients with Rheumatoid Arthritis: A Systematic Review and Meta-Analysis. |
| Risk Factors | The Effect of Statins on Intraocular Pressure and on the Incidence and Progression of Glaucoma: A Systematic Review and Meta-Analysis. |
| Risk Factors | A Meta-Analysis of Glaucoma Risk in Hyperlipidemic Individuals: A Critical Problem in Design. |
| Risk Factors | The effect of smoking on the risk of primary open-angle glaucoma: an updated meta-analysis of six observational studies. [Review] |
| Risk Factors | Circadian Variation in Arterial Blood Pressure and Glaucomatous Optic Neuropathy--A Systematic Review and Meta-Analysis. |
| Risk Factors | Association between glaucoma and obstructive sleep apnea syndrome: a meta-analysis and systematic review. |
| Risk Factors | Diabetes, fasting glucose, and the risk of glaucoma: a meta-analysis. |
| Risk Factors | The Relationship Between Helicobacter pylori Infection and Open-Angle Glaucoma: A Meta-Analysis. [Review] |
| Treatment | Eliciting preferences in glaucoma management-a systematic review of stated-preference studies. |
| Treatment | Clinical implication of recent randomized control trial in primary angle-closure disease management. [Review] |
| Treatment Laser | A Comparison of the 360degree Versus 180degree of Selective Laser Trabeculoplasty (SLT) in the Treatment of Open Angle Glaucoma (OAG) and Ocular Hypertension (OHT): A Comprehensive Systematic Review and Meta-Analysis. |
| Treatment-IVI | Trabeculectomy Augmented With Anti-VEGF Improves Surgical Outcomes in Glaucoma: A Systematic Review and Meta-Analysis. |
| Treatment-IVI | Comparative efficacy and safety of different anti-VEGF agents combined with different delivery methods for neovascular glaucoma: a systematic review and Bayesian network meta-analysis. |
| Treatment-IVI | Anti-vascular endothelial growth factor for neovascular glaucoma. |
| Treatment-IVI | Effect of anti-vascular endothelial growth factor on the surgical outcome of neovascular glaucoma: An overview and meta-analysis. |
| Treatment-IVI | Safety of antivascular endothelial growth factor administration in the ocular anterior segment in pterygium and neovascular glaucoma treatment: Systematic review and meta-analysis. |
| Treatment-IVI | The Effects of Bevacizumab in Augmenting Trabeculectomy for Glaucoma: A Systematic Review and Meta-Analysis of Randomized Controlled Trials. |
| Treatment-IVI | Anti-vascular endothelial growth factor for control of wound healing in glaucoma surgery. |
| Treatment-IVI | Beneficial effects of adjuvant intravitreal bevacizumab injection on outcomes of Ahmed glaucoma valve implantation in patients with neovascular glaucoma: systematic literature review. |
| Treatment-Laser | Visual Acuity Outcomes After Trans-scleral Cyclophotocoagulation: A Systematic Review and Meta-analysis. |
| Treatment-Laser | Is Selective Laser Trabeculoplasty Effective in Reducing Intraocular Pressure for Primary Angle-Closure Glaucoma? A Systematic Review and Meta-Analysis. |
| Treatment-Laser | Noninferiority of selective and micropulse laser trabeculoplasties: a meta-analysis and systematic review. |
| Treatment-Laser | Dysphotopsia and location of laser iridotomy: a systematic review. |
| Treatment-Laser | Efficacy and safety of transscleral cyclophotocoagulation versus cyclocryotherapy in the treatment of intractable glaucoma: A systematic review and meta-analysis. |
| Treatment-Laser | 180- Versus 360-Degree Selective Laser Trabeculoplasty in Open Angle Glaucoma and Ocular Hypertension: A Systematic Review and Meta-Analysis. |
| Treatment-Laser | Anti-inflammatory treatment after selective laser trabeculoplasty: a systematic review of the literature and meta-analysis of randomized control trials. |
| Treatment-Laser | The efficacy and safety of micropulse transscleral laser treatment in glaucoma: a systematic review and meta-analysis. |
| Treatment-Laser | Iridotomy to slow progression of visual field loss in angle-closure glaucoma. |
| Treatment-Laser | Selective laser trabeculoplasty is safe and effective in patients previously treated with prostaglandin analogs: An evidence-based review. [Review] |
| Treatment-Laser | Laser trabeculoplasty for open-angle glaucoma and ocular hypertension. |
| Treatment-Laser | Application of Nd:YAG laser to the anterior vitreous in malignant glaucoma - a systemic review and meta-analysis. |
| Treatment-Laser | The effectiveness of AGV, Ex-PRESS, or trabeculectomy in the treatment of primary and secondary glaucoma: a systematic review and network meta-analysis. |
| Treatment-Laser | Effects of anti-inflammatory treatment on efficacy of selective laser trabeculoplasty: a systematic review and meta-analysis. |
| Treatment-Laser | Surgical and laser interventions for pseudoexfoliation glaucoma systematic review of randomized controlled trials. |
| Treatment-Laser | Laser Trabeculoplasty for Open-Angle Glaucoma: A Systematic Review and Network Meta-Analysis. |
| Treatment-Laser | Laser peripheral iridoplasty for chronic angle closure. |
| Treatment-Laser | Cyclodestructive procedures for refractory glaucoma. |
| Treatment-Laser | Endoscopic cyclophotocoagulation (ECP) for open angle glaucoma and primary angle closure. |
| Treatment-Laser | Iridotomy to slow progression of visual field loss in angle-closure glaucoma. |
| Treatment-Laser | Cyclodestructive procedures for non-refractory glaucoma. |
| Treatment-Laser | Peripheral iridotomy for pigmentary glaucoma. |
| Treatment-Laser | Systematic review and meta-analysis on the efficacy of selective laser trabeculoplasty in open-angle glaucoma. |
| Treatment-Laser | Selective laser trabeculoplasty compared with medical treatment for the initial management of open angle glaucoma or ocular hypertension. |
| Treatment-Medical | Efficacy and Drug Interactions of Glaucoma Medications: A Systematic Review and Component Network Meta-analysis. |
| Treatment-Medical | Protocol for a systematic review and network meta-analysis comparing the efficacy and safety of benzalkonium chloride-preserved, alternatively preserved and preservative-free eyedrops in the treatment of glaucoma. |
| Treatment-Medical | Treatment of glaucoma with drug-loaded contact lenses: A systematic review and meta-analysis. |
| Treatment-Medical | The Effect of Prostaglandin Analogs on Central Corneal Thickness of Patients with Glaucoma or Ocular Hypertension: A Systematic Review. |
| Treatment-Medical | Comparison of Netarsudil/Latanoprost Therapy with Latanoprost Monotherapy for Lowering Intraocular Pressure: A Systematic Review and Meta-analysis. |
| Treatment-Medical | A scoping review and network meta-analysis for efficacy and safety of glaucoma medication in Japanese patients. |
| Treatment-Medical | Efficacy of bimatoprost for the treatment of primary open-angle glaucoma: A protocol of systematic review and meta-analysis. |
| Treatment-Medical | Evaluation of Generic versus Original Prostaglandin Analogues in the Treatment of Glaucoma: A Systematic Review and Meta-Analysis. |
| Treatment-Medical | Uveitis and cystoid macular oedema secondary to topical prostaglandin analogue use in ocular hypertension and open angle glaucoma. |
| Treatment-Medical | Side effects of acetazolamide: a systematic review and meta-analysis assessing overall risk and dose dependence. |
| Treatment-Medical | Efficacy and safety of prostaglandin analogues in primary open-angle glaucoma or ocular hypertension patients: A meta-analysis. |
| Treatment-Medical | Efficacy of travoprost for the treatment of patients with glaucoma. |
| Treatment-Medical | Steroids and nonsteroidal anti-inflammatory drugs in the postoperative regime after trabeculectomy - which provides the better outcome? A systematic review and meta-analysis. |
| Treatment-Medical | The Effect of Vitamins on Glaucoma: A Systematic Review and Meta-Analysis. |
| Treatment-Medical | Safety and Efficacy of Omidenepag Isopropyl for Elevated Intraocular Pressure: A Systematic Review and Meta-Analysis. |
| Treatment-Medical | Evaluation of the nutraceutical Palmitoylethanolamide in reducing intraocular pressure (IOP) in patients with glaucoma or ocular hypertension: a systematic review and meta-analysis. |
| Treatment-Medical | Comparative analysis of medical treatments for long-term control of normal tension glaucoma: A systematic review and model-based network meta-analysis. |
| Treatment-Medical | Selective Laser Trabeculoplasty Versus Medical Therapy for the Treatment of Open Angle Glaucoma or Ocular Hypertension: A Systematic Review and Meta-Analysis of Randomized Controlled Trials. |
| Treatment-Medical | Clinical Efficacy of Omidenepag Isopropyl for Primary Open-Angle Glaucoma, Normal Tension Glaucoma, or Ocular Hypertension: A Meta-Analysis. |
| Treatment-Medical | Efficacy and safety of Fufang Xueshuantong Capsules in combination with conventional drugs in the treatment of glaucoma: A meta-analysis and trial sequential analysis. |
| Treatment-Medical | Effect of prostaglandin analogues on central corneal thickness in patients with glaucoma: A systematic review and meta-analysis with trial sequential analysis. |
| Treatment-Medical | Short-term efficacy of latanoprostene bunod for the treatment of open-angle glaucoma and ocular hypertension: a systematic literature review and a network meta-analysis. |
| Treatment-Medical | Intraocular pressure-lowering efficacy and ocular safety of Rho-kinase inhibitor in glaucoma: a meta-analysis and systematic review of prospective randomized trials. |
| Treatment-Medical | Comparative efficacy and safety of preserved versus preservative-free beta-blockers in patients with glaucoma or ocular hypertension: a systematic review. |
| Treatment-Medical | The efficacy of the fixed combination of latanoprost and timolol versus other fixed combinations for primary open-angle glaucoma and ocular hypertension: A systematic review and meta-analysis. |
| Treatment-Medical | Can neuroprotection effectively manage primary open-angle glaucoma? a protocol of systematic review and meta-analysis. |
| Treatment-Medical | The influence of topical non-steroidal anti-inflammatory drugs on the intraocular pressure lowering effect of topical prostaglandin analogues-A systemic review and meta-analysis. |
| Treatment-Medical | Can dorzolamide/timolol-fixed combination effectively treat primary open-angle glaucoma?: A protocol for systematic review and meta-analysis. |
| Treatment-Medical | Impact of nutraceuticals on glaucoma: A systematic review. |
| Treatment-Medical | Evidence on the neuroprotective properties of brimonidine in glaucoma. |
| Treatment-Medical | Topical medical therapy and ocular perfusion pressure in open angle glaucoma: a systematic review and meta-analysis. |
| Treatment-Medical | Efficacy and safety of different regimens for primary open-angle glaucoma or ocular hypertension: a systematic review and network meta-analysis. |
| Treatment-Medical | Therapeutic efficacy of latanoprost on primary open angle glaucoma: A protocol for a systematic review of randomized controlled trial. |
| Treatment-Medical | Missed opportunity from randomised controlled trials of medical interventions for open-angle glaucoma. |
| Treatment-Medical | Perioperative medications for preventing temporarily increased intraocular pressure after laser trabeculoplasty. |
| Treatment-Medical | Neuroprotection for treatment of glaucoma in adults. |
| Treatment-Medical | Comparative Effectiveness of First-Line Medications for Primary Open-Angle Glaucoma: A Systematic Review and Network Meta-analysis. |
| Treatment-Medical | Sustained Elevation of Intraocular Pressure Associated with Intravitreal Administration of Anti-vascular Endothelial Growth Factor: A Systematic Review and Meta-Analysis. |
| Treatment-Medical | Meta-analysis of the Efficacy and Safety of Latanoprost Monotherapy in Patients With Angle-closure Glaucoma. |
| Treatment-Medical | The effect of flavonoids on visual function in patients with glaucoma or ocular hypertension: a systematic review and meta-analysis. |
| Treatment-Medical | Efficacy and tolerability of prostaglandin-timolol fixed combinations: an updated systematic review and meta-analysis. |
| Treatment-Medical | A model-based dose-response meta-analysis of ocular hypotensive agents as a drug development tool to evaluate new therapies in glaucoma. [Review] |
| Treatment-Medical | Meta-analysis of randomized controlled trials comparing latanoprost with other glaucoma medications in chronic angle-closure glaucoma. |
| Treatment-Medical_Paed | Topical Netarsudil in Childhood Glaucoma: A Systematic Review. |
| Treatment-Medical-Paed | Medical management of pediatric glaucoma: lessons learned from randomized clinical trials. |
| Treatment-Non Pharmacological | Effects of non-pharmacological interventions on anxiety, depression, and sleep quality in patients with postoperative glaucoma: A protocol for systematic review and network meta-analysis. |
| Treatment-Other | The effect of continuous positive airway pressure therapy on intraocular pressure in patients with OSA: a systematic review and meta-analysis. |
| Treatment-Other | Efficacy of Ginkgo biloba on parameters in glaucoma: A systematic review. |
| Treatment-Other | Effect of various relaxation techniques on the intraocular pressure of patients with glaucoma: systematic review and meta-analysis. |
| Treatment-Other | Efficacy of citicoline as a supplement in glaucoma patients: A systematic review. |
| Treatment-Other | Effect of yoga on intra-ocular pressure in patients with glaucoma: A systematic review and meta-analysis. |
| Treatment-Other | Carotenoids in the Management of Glaucoma: A Systematic Review of the Evidence. |
| Treatment-Other | Acupuncture for glaucoma. |
| Treatment-Other | The effect of Bushen Huoxue method in treating glaucoma: A protocol for systematic review and meta-analysis. |
| Treatment-Other | Nutritional supplementation in the treatment of glaucoma: A systematic review. |
| Treatment-Other | Effectiveness of multiple therapeutic strategies in neovascular glaucoma patients: A PRISMA-compliant network meta-analysis. |
| Treatment-Other | Do cannabinoids play a role in the control of glaucoma?. [Review] [Spanish, English] |
| Treatment-Outcomes-Paed | Outcome measures in childhood glaucoma: a systematic review of randomized controlled trials. |
| Treatment-Surgery | What is the long-term efficacy and safety of selective laser trabeculoplasty in the management of primary open-angle glaucoma? A systematic review and meta-analysis. [Review] |
| Treatment-Surgery | The comparative efficacy and safety of one-site versus two-site phacotrabeculectomy: an updated systematic review and meta-analysis. |
| Treatment-Surgery | Intraoperative Injection Versus Sponge-Applied Mitomycin C During Trabeculectomy In Glaucoma: A Systematic Review and Meta-Analysis. |
| Treatment-Surgery | Outcomes of Baerveldt implantation for pediatric glaucoma following surgery: a systematic review and meta-analysis. |
| Treatment-Surgery | The comparative efficacy and safety of one-site versus two-site phacotrabeculectomy: an updated systematic review and meta-analysis. |
| Treatment-Surgery | Standalone Canaloplasty and Trabeculotomy Using the OMNI Surgical System in Patients With Open Angle Glaucoma: A Systematic Review and Meta-analysis. |
| Treatment-Surgery | Intraoperative Injection Versus Sponge-Applied Mitomycin C During Trabeculectomy In Glaucoma: A Systematic Review and Meta-Analysis. |
| Treatment-Surgery | The Paul Glaucoma Implant: a systematic review of safety, efficacy, and emerging applications. [Review] |
| Treatment-Surgery | Efficacy of Canaloplasty for the Management of Primary Open-Angle Glaucoma: Protocol for a Systematic Review. |
| Treatment-Surgery | Comparison of Methods to Deliver Mitomycin C in Trabeculectomy Surgery: A Systematic Review and Meta-Analysis. [Review] |
| Treatment-Surgery | Efficacy and complications of microcatheter-assisted minimally invasive glaucoma surgery for open-angle glaucoma: A systematic review and network meta-analysis. |
| Treatment-Surgery | Ahmed and baerveldt in glaucoma surgery: what is the safest choice? - a systematic review and meta-analysis. |
| Treatment-Surgery | Does standalone phacoemulsification lower intraocular pressure in glaucomatous eyes? A systematic review and meta-analysis. |
| Treatment-Surgery | Trabeculectomy Bleb Characteristics in Relation to Bleb Success Using Anterior Segment Optical Coherence Tomography-A Systematic Review and Meta-Analysis. |
| Treatment-Surgery | Micropulse Transscleral Laser Treatment Versus Continuous Wave Transscleral Cyclophotocoagulation for the Treatment of Glaucoma or Ocular Hypertension: A Meta-Analysis. |
| Treatment-Surgery | Visual field outcomes after cataract surgery with or without microinvasive glaucoma surgery in open-angle glaucoma: a systematic review and meta-analysis of RCTs reporting visual field results. |
| Treatment-Surgery | Minimally Invasive Glaucoma Surgery. [Review] |
| Treatment-Surgery | Efficacy analysis of microinvasive glaucoma surgery alone or in combination with phacoemulsification in patients with normal tension glaucoma: a systematic review and meta-analysis. |
| Treatment-Surgery | PreserFlo MicroShunt versus trabeculectomy: an updated meta-analysis and systematic review. |
| Treatment-Surgery | Safety and efficacy of laser trabeculoplasty compared to drug therapy for the management of open-angle glaucoma: systematic review and meta-analysis study. |
| Treatment-Surgery | Phacoviscocanalostomy versus phacotrabeculectomy to treat glaucoma associated with cataracts: a meta-analysis. |
| Treatment-Surgery | Phacoemulsification combined with trabecular meshwork-Schlemm canal-based minimally invasive glaucoma surgery in primary angle-closure glaucoma: a systematic review and meta-analysis. |
| Treatment-Surgery | Ahmed glaucoma valve implant for refractory glaucoma in children: A systematic review and meta-analysis. |
| Treatment-Surgery | A systematic review and network meta-analysis of surgical interventions for glaucoma following penetrating keratoplasty. |
| Treatment-Surgery | Impact of Glaucoma Surgery on Corneal Graft Survival After Keratoplasty: A Systematic Review and Meta-Analysis. |
| Treatment-Surgery | Combined Microinvasive Glaucoma Surgery With Phacoemulsification in Open-Angle Glaucoma: A Systematic Review and Meta-analysis. |
| Treatment-Surgery | Comparative efficacies of 13 surgical interventions for primary congenital glaucoma in children: a network meta-analysis of randomized clinical trials. |
| Treatment-Surgery | Effectiveness and Safety of Trabeculectomy Versus Tube Shunt Implantation for Uveitic Glaucoma: A Systematic Review and Meta-Analysis. |
| Treatment-Surgery | Efficacy of XEN gel stent implantation combined with mitomycin C injection in the treatment of open-angle glaucoma: a meta-analysis. |
| Treatment-Surgery | Efficacy and safety of intraoperative injection of mitomycin C during trabeculectomy: a systematic review and meta-analysis. |
| Treatment-Surgery | Non-penetrating filtration surgery versus trabeculectomy in postoperative astigmatism: a meta-analysis. |
| Treatment-Surgery | Angle-based minimally invasive glaucoma surgery in normal tension glaucoma: A systematic review and meta-analysis. |
| Treatment-Surgery | One-Year Outcomes of Preserflo Microshunt for Primary Open Angle Glaucoma: A Systematic Review and Meta-Analysis. |
| Treatment-Surgery | Does Bevacizumab Improve Outcomes After Ahmed Glaucoma Valve Implantation for Refractory Glaucoma?: A Meta-Analysis. |
| Treatment-Surgery | Effect of mitomycin C and 5-fluorouracil on wound healing in patients undergoing glaucoma surgery: A meta-analysis. |
| Treatment-Surgery | The Effect of Phacoemulsification on the Intraocular Pressure of Patients With Open Angle Glaucoma: A Systematic Review and Meta-Analysis. |
| Treatment-Surgery | Hypotony Failure Criteria in Glaucoma Surgical Studies and Their Influence on Surgery Success. |
| Treatment-Surgery | The efficacy of adjunctive mitomycin C and/or anti-VEGF agents on glaucoma tube shunt drainage device surgeries: a systematic review. |
| Treatment-Surgery | Comparison between MIGS with trabeculectomy in the management of open-angle glaucoma with cataract: A systematic review. |
| Treatment-Surgery | Comparing the safety and efficacy of Preserflo Microshunt implantation and trabeculectomy for glaucoma: A systematic review and meta-analysis. |
| Treatment-Surgery | Minimally Invasive Bleb Surgery for Glaucoma: A Health Technology Assessment. |
| Treatment-Surgery | Phacoemulsification combined with goniosynechialysis versus phacoemulsification alone for patients with primary angle-closure disease: A meta-analysis. |
| Treatment-Surgery | Two-Year Performance and Safety Results of the MINIject Supraciliary Implant in Patients With Primary Open-Angle Glaucoma: Meta-Analysis of the STAR-I, II, III Trials. |
| Treatment-Surgery | Comparison of Repeat Trabeculectomy Versus Ahmed Valve Implantation After Initial Failed Trabeculectomy Surgery. [Review] |
| Treatment-Surgery | Short-Term Outcomes of Trabeculectomy With or Without Anti-VEGF in Patients With Neovascular Glaucoma: A Systematic Review and Meta-Analysis. |
| Treatment-Surgery | Phacoemulsification with and without iStent: A Systematic Review and Meta-Analysis of Comparative Studies. |
| Treatment-Surgery | Efficacy of Ahmed and Baerveldt glaucoma drainage device implantation in the pediatric population: A systematic review and meta-analysis. |
| Treatment-Surgery | Complications and post-operative interventions in XEN45 gel stent implantation in the treatment of open angle glaucoma-a systematic review and meta-analysis. |
| Treatment-Surgery | Comparative efficacies of 13 surgical interventions for primary congenital glaucoma in children: a network meta-analysis of randomized clinical trials. |
| Treatment-Surgery | Comparison of tube shunt implantation and trabeculectomy for glaucoma: a systematic review and meta-analysis. |
| Treatment-Surgery | Comparison of Aurolab Aqueous Drainage Implant with Ahmed Glaucoma Valve for Refractory Glaucoma: A Meta-Analysis. |
| Treatment-Surgery | Combined Surgery Versus Phacoemulsification Alone for Patients with Primary Angle-Closure Glaucoma: A meta-analysis. |
| Treatment-Surgery | Gonioscopy Assisted Transluminal Trabeculotomy:A Boon to Developing Nations-A Systematic Review. |
| Treatment-Surgery | Comparison of Hydrus and iStent microinvasive glaucoma surgery implants in combination with phacoemulsification for treatment of open-angle glaucoma: systematic review and network meta-analysis. |
| Treatment-Surgery | Comparisons of the short-term effectiveness and safety of surgical treatment for neovascular glaucoma: a systematic review and network meta-analysis. |
| Treatment-Surgery | Protocol for systematic review and network meta-analysis of comparative effectiveness of surgical interventions for primary congenital glaucoma. |
| Treatment-Surgery | Differences in the Surgical Outcomes of Glaucoma Surgery in Patients of African Caribbean Descent. |
| Treatment-Surgery | Meta-Analysis of the Comprehensive Efficacy of Intraocular Lens Implantation in Glaucoma Patients. |
| Treatment-Surgery | Systematic Review of MIGS and Non-Penetrating Glaucoma Procedures for Uveitic Glaucoma. |
| Treatment-Surgery | The efficacy of XEN gel stent implantation in glaucoma: a systematic review and meta-analysis. |
| Treatment-Surgery | Microstent Implantation with Phacoemulsification Versus Phacoemulsification Alone for Patients with Open Angle Glaucoma: A Systematic Review and Meta-Analysis of Randomised Clinical Trials. |
| Treatment-Surgery | Reintervention rate in glaucoma filtering surgery: A systematic review and meta-analysis. |
| Treatment-Surgery | Corneal Endothelial Cell Density Loss after Glaucoma Surgery Alone or in Combination with Cataract Surgery: A Systematic Review and Meta-analysis. |
| Treatment-Surgery | Standalone XEN45 Gel Stent implantation in the treatment of open-angle glaucoma: A systematic review and meta-analysis. |
| Treatment-Surgery | Noninferiority of Microhook to Trabectome: Trabectome versus Ab Interno Microhook Trabeculotomy Comparative Study (Tram Trac Study). |
| Treatment-Surgery | Postoperative complications in glaucoma surgery: literature review-based recommendations to improve reporting consistency. [Review] |
| Treatment-Surgery | Fornix-based versus limbal-based conjunctival trabeculectomy flaps for glaucoma. |
| Treatment-Surgery | Adjunctive modulation of wound healing during cataract surgery to promote survival of a previous trabeculectomy. |
| Treatment-Surgery | Minimally Invasive Glaucoma Surgical Techniques for Open-Angle Glaucoma: An Overview of Cochrane Systematic Reviews and Network Meta-analysis. [Review] |
| Treatment-Surgery | Standalone XEN45 Gel Stent implantation versus combined XEN45-phacoemulsification in the treatment of open angle glaucoma-a systematic review and meta-analysis. |
| Treatment-Surgery | Corneal endothelial cell density loss following glaucoma surgery alone or in combination with cataract surgery: a systematic review protocol. |
| Treatment-Surgery | Ab interno supraciliary microstent surgery for open-angle glaucoma. |
| Treatment-Surgery | Lens extraction for chronic angle-closure glaucoma. |
| Treatment-Surgery | Standalone iStent Trabecular Micro-bypass Glaucoma Surgery: A Systematic Review and Meta-Analysis. |
| Treatment-Surgery | Ab interno trabecular bypass surgery with Trabectome for open-angle glaucoma. |
| Treatment-Surgery | Estimation of risk-benefit ratio and comparison of post-operative efficacy results between trabeculectomy and canaloplasty. |
| Treatment-Surgery | Ab interno trabecular bypass surgery with Schlemm's canal microstent (Hydrus) for open angle glaucoma. |
| Treatment-Surgery | Selective laser trabeculoplasty versus medication for open-angle glaucoma: systematic review and meta-analysis of randomised clinical trials. |
| Treatment-Surgery | Efficacy and safety evaluation of benzalkonium chloride preserved eye-drops compared with alternatively preserved and preservative-free eye-drops in the treatment of glaucoma: a systematic review and meta-analysis. |
| Treatment-Surgery | A Meta-analysis on the Outcome of Sequential Glaucoma Drainage Implantation. |
| Treatment-Surgery | Treatment of Uveitis and Outcomes of Glaucoma Drainage Implant Surgery: A Meta-Analysis. |
| Treatment-Surgery | Canaloplasty for the treatment of primary open-angle glaucoma: A protocol of systematic review and meta-analysis. |
| Treatment-Surgery | Needling after trabeculectomy - does augmentation by anti-metabolites provide better outcomes and is Mitomycin C better than 5-Fluoruracil? A systematic review with network meta-analyses. |
| Treatment-Surgery | Overview of cicatricial modulators in glaucoma fistulizing surgery. |
| Treatment-Surgery | Microcatheter-Assisted Circumferential Trabeculotomy versus Conventional Trabeculotomy for the Treatment of Childhood Glaucoma: A Meta-analysis. [Review] |
| Treatment-Surgery | Minimally Invasive Glaucoma Surgery: Where Is the Evidence?. |
| Treatment-Surgery | Systematic Review and Meta-Analysis of Comparing Phacoemulsification Combined with goniosynechialysis to other mainstream procedures in treating patients with angle-closure glaucoma. |
| Treatment-Surgery | Ologen implant versus mitomycin-C for trabeculectomy: A meta-analysis. |
| Treatment-Surgery | Aqueous shunts with mitomycin C versus aqueous shunts alone for glaucoma. |
| Treatment-Surgery | Ab interno trabecular bypass surgery with iStent for open-angle glaucoma. |
| Treatment-Surgery | Efficacy of glaucoma drainage devices in uveitic glaucoma and a meta-analysis of the literature. [Review] |
| Treatment-Surgery | Influence of glaucoma surgery on visual function: a clinical cohort study and meta-analysis. [Review] |
| Treatment-Surgery | The role of phacoemulsification in glaucoma therapy: A systematic review and meta-analysis. |
| Treatment-Surgery | Comparison of pars plana with anterior chamber glaucoma drainage device implantation for glaucoma: a meta-analysis. [Review] |
| Treatment-Surgery | Efficacy and Safety of Bevacizumab Combined with Mitomycin C or 5-Fluorouracil in Primary Trabeculectomy: A Meta-Analysis of Randomized Clinical Trials. [Review] |
| Treatment-Surgery | Is mytomicyn better than 5-fluorouracil as antimetabolite in trabeculectomy for glaucoma?. [Review] [Spanish, English] |
| Treatment-Surgery | Minimally-invasive glaucoma surgeries (MIGS) for open angle glaucoma: A systematic review and meta-analysis. |
| Treatment-Surgery | Aqueous shunts for glaucoma. |
| Treatment-Surgery | Incidence and risk factors for post-penetrating keratoplasty glaucoma: A systematic review and meta-analysis. |
| Treatment-Surgery | The Effects of Phacoemulsification on Intraocular Pressure and Topical Medication Use in Patients With Glaucoma: A Systematic Review and Meta-analysis of 3-Year Data. |
| Treatment-Surgery | Fornix-Based Versus Limbal-Based Conjunctival Trabeculectomy Flaps for Glaucoma: Findings From a Cochrane Systematic Review. |
| Treatment-Surgery | Is device-modified trabeculectomy better than classic surgery for treatment of glaucoma?. [Spanish, English] |
| Treatment-Surgery | Safety and efficacy of canaloplasty versus trabeculectomy in treatment of glaucoma. |
| Treatment-Surgery | Ab interno trabecular bypass surgery with Trabectome for open angle glaucoma. |
| Treatment-Surgery | Clinical Outcomes of Ahmed Glaucoma Valve Implantation With or Without Intravitreal Bevacizumab Pretreatment for Neovascular Glaucoma: A Systematic Review and Meta-Analysis. |
| Treatment-Surgery | The Ahmed shunt versus the Baerveldt shunt for refractory glaucoma: a meta-analysis. [Review] |
| Treatment-Surgery | Lowering of Intraocular Pressure After Phacoemulsification in Primary Open-Angle and Angle-Closure Glaucoma: A Bayesian Analysis. [Review] |
| Treatment-Surgery | Review and meta-analysis of ab-interno trabeculectomy outcomes. [Review] |
| Treatment-Surgery | Fornix-based versus limbal-based conjunctival trabeculectomy flaps for glaucoma. |
| Treatment-Surgery | Mitomycin C versus 5-Fluorouracil for wound healing in glaucoma surgery. |
| Treatment-Surgery | Combined surgery versus cataract surgery alone for eyes with cataract and glaucoma. |
| Treatment-Surgery | Comparison of Ahmed glaucoma valve implantation and trabeculectomy for glaucoma: a systematic review and meta-analysis. |
| Treatment-Surgery | Efficacy of Additional Glaucoma Drainage Device Insertion in Refractory Glaucoma: Case Series with a Systematic Literature Review and Meta-Analysis. |
| Treatment-Surgery | Comparison of the Ahmed glaucoma valve with the Baerveldt glaucoma implant: a meta-analysis. |
| Treatment-Surgery | Comparison of Efficacy Between Endoscopic Cyclophotocoagulation and Alternative Surgeries in Refractory Glaucoma: A Meta-analysis. [Review] |
| Treatment-Surgery | Meta-analysis of selective laser trabeculoplasty versus topical medication in the treatment of open-angle glaucoma. |
| Treatment-Surgery | iStent with Phacoemulsification versus Phacoemulsification Alone for Patients with Glaucoma and Cataract: A Meta-Analysis. |
| Treatment-Surgery | iStent as a Solo Procedure for Glaucoma Patients: A Systematic Review and Meta-Analysis. |
| Treatment-Surgery | Efficacy and Safety of Ologen Implant Versus Mitomycin C in Primary Trabeculectomy: A Meta-analysis of Randomized Clinical Trials. |
| Treatment-Surgery | Efficacy of anti-vascular endothelial growth factor and mitomycin C on wound healing after trabeculectomy in glaucoma patients: A meta-analysis. |
| Treatment-Surgery-OCT | Intra-operative optical coherence tomography in glaucoma surgery-a systematic review. |
| Treatment-Surgery-Paed | Surgical interventions for primary congenital glaucoma. |
| Treatment-Surgery-Paed | Surgical interventions for primary congenital glaucoma. |
| Treatment-Surgical | Efficacy and Safety of Trabeculectomy Versus Nonpenetrating Surgeries in Open-angle Glaucoma: A Meta-analysis. |
| Treatment-Surgical | Surgical treatment of neovascular glaucoma: a systematic review and meta-analysis. |
| Treatment-Surgery | Efficacy and Safety of Different Types of Intraocular Pressure-Lowering Surgeries in Patients with Primary Angle Closure (PAC) or PAC Glaucoma: Systematic Review and Network Meta-Analysis of Randomized Clinical Trials. |

**Supplementary File 3 - Figure 1. Tree map showing absolute number of systematic reviews in each evidence domain**

**Supplementary File 4 - Table 1. Summary of Cochrane Reviews 2015 to 2025**

| **Author, Year** | **Review Title** | **Conclusions (Author's Conclusions in Abstract)** |
| --- | --- | --- |
| **1.Michelessi et al., 2015** | **Optic nerve head and fibre layer imaging for diagnosing glaucoma** | The accuracy of imaging tests for detecting manifest glaucoma was variable across studies, but overall similar for different devices. Accuracy may have been overestimated due to the case-control design, which is a serious limitation of the current evidence base. We recommend that further diagnostic accuracy studies are carried out on patients selected consecutively at a defined step of the clinical pathway, providing a description of risk factors leading to referral and bearing in mind the consequences of false positives and false negatives in the setting in which the diagnostic question is made. Future research should report accuracy for each threshold of these continuous measures or publish raw data. |
| **2.Cabourne et al., 2015** | **Mitomycin C versus 5-Fluorouracil for wound healing in glaucoma surgery** | We found low quality evidence that MMC may be more effective in achieving longer term lower intraocular pressure than 5FU. Further comparative research on MMC and 5FU is needed to enhance reliability and validity of the results shown in this review. Furthermore, the development of new agents that control postoperative scar tissue formation without side effects would be valuable and is justified by the results of this review. |
| **3.Zhang et al., 2015** | **Combined surgery versus cataract surgery alone for eyes with cataract and glaucoma** | There is low quality evidence that combined cataract and glaucoma surgery may result in better IOP control at one year compared with cataract surgery alone. The evidence was uncertain in terms of complications from the surgeries. Furthermore, this Cochrane review has highlighted the lack of data regarding important measures of the patient experience, such as visual field tests, quality of life measurements, and economic outcomes after surgery, and long-term outcomes (five years or more). Additional higher quality RCTs measuring clinically meaningful and patient important outcomes are required to provide evidence to support treatment recommendations. |
| **4.Cheng et al., 2016** | **Anti-Vascular endothelial growth factor for control of wound healing in glaucoma surgery** | The evidence is currently of low quality which is insufficient to refute or support anti-VEGF subconjunctival injection for control of wound healing in glaucoma surgery. The effect on IOP control of anti-VEGF agents in glaucoma patients undergoing trabeculectomy is still uncertain, compared to MMC. Further RCTs of anti-VEGF subconjunctival injection in glaucoma surgery are required, particularly compared to sham treatment with at least 12 months follow-up. |
| **5.Michelessi et al., 2016** | **Peripheral iridotomy for pigmentary glaucoma** | We found insufficient evidence of high quality on the effectiveness of peripheral iridotomy for pigmentary glaucoma or pigment dispersion syndrome. Although adverse events associated with peripheral iridotomy may be minimal, the longer-term effects on visual function and other patient important outcomes have not been established. Future research on this topic should focus on outcomes that are important to patients and the optimal timing of treatment in the disease process (eg, pigment dispersion syndrome with normal IOP, pigment dispersion syndrome with established ocular hypertension, pigmentary glaucoma). |
| **6.Zhang et al., 2017** | **Perioperative medications for preventing temporarily increased intraocular pressure after laser trabeculoplasty** | Perioperative medications are superior to no medication or placebo to prevent IOP spikes during the first two hours and up to 24 hours after LTP, but some medications can cause temporary conjunctival blanching, a shorter-term cosmetic effect. Overall, perioperative treatment was well tolerated and safe. Alpha-2 agonists are useful in helping to prevent IOP increases after LTP, but it is unclear whether one medication in this class of drugs is better than another. There was no notable difference between apraclonidine and pilocarpine in the outcomes we were able to assess. Future research should include participants who have been using these antiglaucoma medications for daily treatment of glaucoma before LTP was performed. |
| **7.Xu et al., 2017** | **Topical medication instillation techniques for glaucoma** | Evidence to evaluate the effectiveness of topical medication instillation techniques for treatment of glaucoma is lacking. It is unclear what, if any, effects instillation techniques have on topical medical therapy for glaucoma. |
| **8.Sena et al., 2017** | **Neuroprotection for treatment of glaucoma in adults** | Although the only trial we included in this review found less visual field loss in the brimonidine treated group, the evidence was of such low certainty that we can draw no conclusions from this finding. Further clinical research is needed to determine whether neuroprotective agents may be beneficial for individuals with OAG. Such research should focus on outcomes important to patients, such as preservation of vision, and how these outcomes relate to cell death and optic nerve damage. As OAG is a chronic, progressive disease with variability in symptoms, RCTs designed to measure the effectiveness of neuroprotective agents require a longer-term follow-up of five years or longer to detect clinically meaningful effects. |
| **9.Tseng et al., 2017** | **Aqueous shunts for glaucoma** | Information was insufficient to conclude whether there are differences between aqueous shunts and trabeculectomy for glaucoma treatment. While the Baerveldt implant may lower IOP more than the Ahmed implant, the evidence was of moderate certainty and it is unclear whether the difference in IOP reduction is clinically significant. Overall, methodology and data quality among existing randomized controlled trials of aqueous shunts was heterogeneous across studies, and there are no well-justified or widely accepted generalizations about the superiority of one surgical procedure or device over another. |
| **10.Michelessi et al., 2018** | **Cyclodestructive procedures for non-refractory glaucoma** | There is insufficient evidence to evaluate the relative effectiveness and safety of cyclodestructive procedures for the primary procedural management of non-refractory glaucoma. Results from the one included trial did not compare cyclophotocoagulation to other procedural interventions and yielded uncertainty about any difference in outcomes when comparing lower energy versus higher energy diode TSCPC. Overall, the effect of laser treatment on IOP control was modest and the number of eyes experiencing vision loss was limited. More research is needed specific to the management of non-refractory glaucoma. |
| **11.King et al., 2018** | **Subconjunctival draining minimally-invasive glaucoma devices for medically uncontrolled glaucoma** | There is currently no higher quality evidence for the effects of subconjunctival draining minimally invasive glaucoma devices for medically uncontrolled open angle glaucoma. Properly designed RCTs are needed to assess the medium and long-term efficacy and safety of this technique. |
| **12.Foo et al., 2019** | **Aqueous shunts with mitomycin C versus aqueous shunts alone for glaucoma** | We found insufficient evidence in this review to suggest MMC provides any postoperative benefit for glaucoma patients who undergo aqueous shunt surgery. Data across all five included trials were sparse and the reporting of study methods required to assess bias was inadequate. Future RCTs of this intervention should report methods in sufficient detail to permit assessment of potential bias and estimate target sample sizes based on clinically meaningful effect sizes. |
| **13.TÃ et al., 2019** | **Endoscopic cyclophotocoagulation (ECP) for open angle glaucoma and primary angle closure** | There is currently no high quality evidence for the effects of ECP for OAG and primary angle closure. Properly designed RCTs are needed to assess the medium and long-term efficacy and safety of this technique. |
| **14.Le et al., 2019** | **Ab interno trabecular bypass surgery with iStent for open-angle glaucoma** | There is very low-quality evidence that treatment with iStent may result in higher proportions of participants who are drop-free or achieving better IOP control, in the short, medium, or long-term. Results from the 13 studies with results not yet available may clarify the benefits of treatment of people with iStent. Additionally, future MIGS studies should consider measuring quality of life and outcomes that reflect people's ability to perform vision-dependent activities. |
| **15.Chen et al., 2019** | **Cyclodestructive procedures for refractory glaucoma** | Evidence from five studies included in this review was inconclusive as to whether cyclodestructive procedures for refractory glaucoma result in better outcomes and fewer complications than other glaucoma treatments, and whether one type of cyclodestructive procedure is better than another. The most commonly reported adverse events across all five studies were hypotony and phthisis bulbi. Large, well-designed randomized controlled trials are needed. Patient reported outcomes such as pain and quality of life should be considered as primary outcomes or important secondary outcomes of future trials. |
| **16.Gagrani et al., 2020** | **Surgical interventions for primary congenital glaucoma** | The evidence suggests that there may be little to no evidence of difference between CTT and routine conventional trabeculotomy, or between visco-trabeculotomy and routine conventional trabeculotomy . A 360-degree circumferential trabeculotomy may show greater surgical success than conventional trabeculotomy. Considering the rarity of the disease, future research would benefit from a multicenter, possibly international trial, involving parents of children with PCG and with a follow-up of at least one year. |
| **17.Law et al., 2020** | **Acupuncture for glaucoma** | At this time, it is impossible to draw reliable conclusions from available data to support the use of acupuncture for treatment of patients with glaucoma. Because of ethical considerations, RCTs comparing acupuncture alone with standard glaucoma treatment or placebo are unlikely to be justified in countries where the standard of care has already been established. |
| **18.Jindal et al., 2020** | **Non contact tests for identifying people at risk of primary angle closure glaucoma** | The finding that LACD performed as well as index tests that use sophisticated imaging technologies, confirms the potential for this test for case-detection of occludable angles in high-risk populations. However, methodological issues across studies may have led to our estimates of test accuracy being higher than would be expected in standard clinical practice. There is still a need for high-quality studies to evaluate the performance of non-invasive tests for angle assessment in both community-based and secondary care settings. |
| **19.Otarola et al., 2021** | **Ab interno trabecular bypass surgery with Schlemm´s canal microstent (Hydrus) for open angle glaucoma** | In people with cataracts and generally mild to moderate OAG, there is moderate certainty evidence that the Hydrus microstent with cataract surgery compared to cataract surgery alone, likely increases the proportion of participants who do not require IOP lowering medication, and may further reduce IOP at short and medium-term follow-up. There is moderate certainty evidence that the Hydrus micro-stent is probably more effective than the iStent in lowering IOP of people with OAG in the short-term. Few studies were available on the effects of the Hydrus microstent, therefore the results of this review may not be applicable to all people with OAG, particularly in selected people with medically uncontrolled glaucoma, since IOP was controlled with medication in many participants in the included studies. Complications may be rare using the Hydrus microstent, as well as the comparator iStent, but larger studies are needed to investigate its safety. |
| **20.Ng et al., 2021** | **Adjunctive modulation of wound healing during cataract surgery to promote survival of a previous trabeculectomy** | There is a need for an RCT to investigate the role of adjuvant wound modulating therapy at the time of cataract surgery to promote survival of a functioning trabeculectomy. |
| **21.Sandhu et al., 2021** | **Ab interno supraciliary microstent surgery for open-angle glaucoma** | Data from this single RCT show superiority of supraciliary microstent surgery when combined with phacoemulsification compared to phacoemulsification alone in achieving medication free control of OAG. However, there are long term safety concerns with the device used in this trial, relating to the observed significant loss of corneal endothelial cells at five years following device implantation. At the time of this review, this device has been withdrawn from the market. This review has found that few high-quality studies exist comparing supraciliary microstent surgery to standard medical, laser or surgical glaucoma treatments. This should be addressed by further appropriately designed RCTs with sufficient long-term follow-up to ensure robust safety data are obtained. Consideration of health-related quality of life outcomes should also feature in trial design. |
| **22.Bayliss et al., 2021** | **Laser peripheral iridoplasty for chronic angle closure** | After reviewing the outcomes of four RCTs, argon LPIp as an intervention may be no more clinically effective than comparators in the management of people with chronic angle closure. Despite a potential positive impact on anterior chamber morphology, its use in clinical practice in treating people with chronic angle closure is not supported by the results of trials published to date. Given these results, further research into LPIp is unlikely to be worthwhile. |
| **23.Theventhiran et al., 2021** | **Fornix based versus limbal based conjunctival trabeculectomy flaps for glaucoma** | The main result of this review was that there was uncertainty as to the difference between fornix and limbal based trabeculectomy surgeries due to the small number of events and confidence intervals that cross the null. This also applied to postoperative complications, but without any impact on long-term failure rate between the two surgical techniques. |
| **24.Ong et al., 2021** | **Lens extraction for chronic angle-closure glaucoma** | Moderate certainty evidence showed that lens extraction has an advantage over LPI in treating chronic PACG with clear crystalline lenses over three years of follow-up; ultimately, the decision for intervention should be part of a shared decision-making process between the clinician and the patient. For people with chronic PACG and visually significant cataracts, low certainty evidence suggested that combining phacoemulsification with either viscogonioplasty or goniosynechialysis does not confer any additional benefit over phacoemulsification alone. There was insufficient evidence to draw any meaningful conclusions regarding phacoemulsification versus trabeculectomy. Low certainty evidence suggested that combining phacoemulsification with trabeculectomy does not confer any additional benefit over phacoemulsification alone, and may cause more complications instead. These conclusions only apply to short to medium term outcomes; studies with longer follow-up periods can help assess whether these effects persist in the long term. |
| **25.Hu et al., 2021** | **Ab interno trabecular bypass surgery with Trabectome for open-angle glaucoma** | There is currently no high-quality evidence for the outcomes of ab interno trabecular bypass surgery with Trabectome for open-angle glaucoma. Properly designed RCTs are needed to assess the long-term efficacy and safety of this technique. |
| **26.Rolim-de-Moura et al., 2022** | **Laser trabeculoplasty for open-angle glaucoma and ocular hypertension** | Laser trabeculoplasty may work better than topical medication in slowing down the progression of open-angle glaucoma (rate of visual field loss) and may be similar to modern eye drops in controlling eye pressure at a lower cost. It is not associated with serious unwanted effects, particularly for the newer types of trabeculoplasty, such as selective laser trabeculoplasty. |
| **27.Clement Freiberg et al., 2022** | **Rho kinase inhibitor for primary open angle glaucoma and ocular hypertension** | The current evidence suggests that in people diagnosed with OHT or (P)OAG, the hypotensive effect of netarsudil may be inferior to latanoprost and slightly inferior to timolol. Combining netarsudil and latanoprost probably further reduces IOP compared with monotherapy. Netarsudil as mono or combination therapy may result in more ocular AEs. However, the certainty of evidence was very low or low for all comparisons except timolol. |
| **28.Park et al., 2023** | **Device modified trabeculectomy for glaucoma** | Use of an ExPRESS plus trabeculectomy may produce greater IOP reduction at one-year follow-up than standard trabeculectomy; however, due to potential biases and imprecision in effect estimates, the certainty of evidence is low. PreserFlo MicroShunt may be inferior to standard trabeculectomy in lowering IOP. However, PreserFlo MicroShunt may prevent postoperative hypotony and bleb leakage. Overall, device-modified trabeculectomy appears associated with a lower risk of cataract surgery within five years compared with standard trabeculectomy. Due to various limitations in the design and conduct of the included studies, the applicability of this evidence synthesis to other populations or settings is uncertain. Further research is needed to determine the effectiveness and safety of other devices in subgroup populations, such as people with different types of glaucoma, of various races and ethnicity, and with different lens types (e.g. phakic, pseudophakic). |
| **29.Rittiphairoj et al., 2023** | **Anti-Vascular endothelial growth factor for neovascular glaucoma** | Anti-VEGFs as an adjunct to conventional treatment could help reduce IOP in NVG in the short term (four to six weeks), but there is no evidence that this is likely in the longer term. Currently available evidence regarding the short- and long-term effectiveness and safety of anti-VEGFs in achieving control of IOP, visual acuity, and complete regression of new iris vessels in NVG is insufficient. More research is needed to investigate the effect of these medications compared with, or in addition to, conventional surgical or medical treatment in achieving these outcomes in NVG. |
| **30.Rouse et al., 2023** | **Iridotomy to slow progression of visual field loss in angle closure glaucoma** | We did not find sufficient evidence to draw any meaningful conclusions on the use of iridotomy for the purpose of slowing progression of visual field loss. No study reported on progressive visual field loss, the primary outcome of this review. Although there is moderate certainty evidence that iridotomy results in improved gonioscopic findings, in is unclear if these findings translate to clinically meaningful benefits. |
| **31.Ong et al., 2023** | **Lens extraction versus laser peripheral iridotomy for acute primary angle closure** | Low certainty evidence suggests that early lens extraction may produce more favorable outcomes compared to initial LPI in terms of IOP control. Evidence for other outcomes is less clear. Future high quality and longer-term studies evaluating the effects of either intervention on the development of glaucomatous damage and visual field changes as well as health-related quality of life measures would be helpful. |
| **32.Kruoch et al., 2023** | **Multifaceted behavioral interventions to improve topical glaucoma therapy adherence in adults** | Based on very low and low certainty evidence identified in this review, it is unclear if multifaceted behavioral interventions have a beneficial effect on patient adherence to topical IOP lowering medications and IOP in people with ocular hypertension or glaucoma. In most cases, we were unable to conduct meta-analysis due to the diversity of interventions, varying outcome definitions, and inconsistent reporting across studies. Future research would benefit from the adoption of standardized measurements and reporting methods for adherence to medication and clinical outcomes on disease stability, while taking social determinants into account. |
| **33.Piyasena et al., 2025** | **Prognostic factors associated with progression of open‐angle glaucoma in adults** | There is moderate‐certainty evidence to support the finding that bilateral disease is a prognostic factor associated with VF progression in people with glaucoma. There is low‐certainty evidence that female sex and the presence of disc hemorrhage are associated with progression, while treatment with pharmacologics is protective of progression. The evidence is uncertain about the associations between progression and all other prognostic factors that we identified. Properly designed prognostic factor research studies are required in the future. |
